# Expression profiling of peripheral blood miRNA using RNAseq technology in dairy cows with *Escherichia coli*-induced mastitis

**DOI:** 10.1038/s41598-018-30518-2

**Published:** 2018-08-23

**Authors:** Zhuo-Ma Luoreng, Xing-Ping Wang, Chu-Gang Mei, Lin-Sen Zan

**Affiliations:** 1 College of Animal Science and Technology, National Beef Cattle Improvement Center, Northwest A & F University, Yangling, Shaanxi China; 2 Key Laboratory of Zoology in Hunan Higher Education, College of Life Science, Hunan University of Arts and Science, Changde, Hunan China

## Abstract

*E*. *coli* is the main causative agent of mastitis in dairy cows, but the mechanism of molecular regulation underlying the occurrence and development of mastitis has not yet been fully elucidated. In this study, an *E*. *coli*-induced mastitis model was created and RNASeq technology was used to measure the miRNA expression profiles at different times post-infection (0, 1, 3, 5, 7 dpi), as well as to screen for differentially expressed miRNA. The results show detection of 2416 miRNAs, including 628 known miRNAs and 1788 newly discovered miRNAs. A total of 200 differentially expressed miRNAs were found at different time points. Bioinformatics analysis showed that these differentially expressed miRNAs may regulate the occurrence and development of mastitis in dairy cows through seven signal transduction pathways, namely cytokine-cytokine receptor interaction, MAPK signaling pathway, chemokine signaling pathway, leukocyte transendothelial migration, T cell receptor signaling pathway, Toll-like receptor signaling pathway, and cell adhesion molecules. In addition, bta-miR-200a, bta-miR-205, bta-miR-122, bta-miR-182 and the newly discovered conservative_15_7229 might be involved in immune process in late stage of *E*. *coli*-induced mastitis. The results of this study lay the foundation for molecular network analysis of mastitis and molecular breeding of dairy cows.

## Introduction

Mastitis is an inflammatory disease caused by pathogenic microorganisms in mammary tissues. The high incidence of mastitis in dairy cows leads to decreased milk production and lowered dairy product quality, which can seriously affect the profitability of dairy farms^1^. *E*. *coli* is one of the most commonly observed pathogens in dairy cow mastitis. The occurrence and development of mastitis are the results of co-regulation by gene networks composed of multiple genes^2^.

MicroRNAs (miRNA) are recently discovered endogenous non-coding RNAs. They bind to their target mRNAs and degrade or downregulate the mRNAs, thereby regulating a number of biological processes such as growth, development and immunity in humans and animals^3–5^. Studies have shown that, after cells receive exogenous or endogenous signals, changes in expression of miRNA form part of the early response. For example, the expression levels of certain miRNAs change rapidly after disease occurs in an organism to control the scope and intensity of disease development^6–8^. Therefore, miRNA expression levels during disease onset or progression can be used as an early diagnostic biomarker of disease^7,9^. However, miRNA research in dairy mastitis is still in its infancy. In recent years, scholars have mainly used mammary tissues or mammary epithelial cells as research materials for miRNA isolation, identification and functional studies^10–13^. There have only been a few reports on blood miRNAs, for example, in the use of high-throughput techniques to detect miRNA expression profiles in clinical mastitis in dairy cows^14,15^ and in the detection of miRNA expression levels in mononuclear cells from dairy cows with mastitis caused by infection with *S*. *uberis*^16^. The changes in expression of blood miRNA in dairy cows caused by *E*. *coli* infection have not been previously reported. Therefore, in this study, RNAseq was used to determine the expression levels of blood miRNA in *E*. *coli*-induced mastitis at different times after infection, in order to provide a basis for the biotherapy, and molecular mechanism behind mastitis in dairy cows.

## Results

### Overview of sequencing data

To investigate the changes in the expression of blood miRNAs during bovine mastitis, we performed deep sequencing of small RNA libraries using the Illumina Hiseq2500 platform. Based on the raw reads, unreliable sequences were removed as described in the method to obtain high quality clean reads (Table 1). In addition, using Bowtie software^17^, we compared the clean reads with the bovine reference genome to obtain mapped small RNA reads. Quality control ensured that Q30 (%) was greater than 94% for each sample and clean data was greater than 11.89 M, indicating reliable sequencing data for subsequent sRNA.

**Table 1:** Overview of miRNA sequencing data.

dpi	Raw reads	Q30 (%)	Clean reads	Mapped Small RNA Reads
0	30358145	94.33	26833388	16812993
1	18604382	97.23	16600883	10672762
3	16922373	94.16	14615093	9504913
5	14437756	97.15	12570752	8096806
7	12858078	97.45	11887296	7747705

### sRNA classification

Bowtie software is a short sequence alignment software and is especially suited for alignment of reads obtained by high-throughput sequencing. Therefore, we used Bowtie software^17^ to align clean reads with the Silva, GtRNAdb, Rfam, and Repbase databases to remove ncRNA and repetitive sequences such as rRNA, tRNA, snRNA, and snoRNA, and obtained the unannotated reads for miRNA sequences (Table 2).

**Table 2:** Distribution of the small RNA among different categories at different times of mastitis induction.

dpi	Clean reads	rRNA	snRNA	snoRNA	tRNA	Repbase	Unannotated	miRNA	Unannotated small RNA
0	26,833,388	639,348	12	181,237	116,546	494,265	25,401,980	21,961,291	3,440,689
	100.00%	2.38%	0.00%	0.68%	0.43%	1.84%	94.67%	81.84%	12.83%
1	16,600,883	518,804	10	123,445	89,050	317,135	15,552,439	13,350,150	2,202,289
	100.00%	3.13%	0.00%	0.74%	0.54%	1.91%	93.68%	80.42%	13.26%
3	14,615,093	501,037	12	109,114	127,350	270,833	13,606,747	11,593,688	2,013,059
	100.00%	3.43%	0.00%	0.75%	0.87%	1.85%	93.10%	79.33%	13.77%
5	12,570,752	362,417	12	99,001	45,138	262,799	11,801,385	10,157,070	1,644,315
	100.00%	2.88%	0.00%	0.79%	0.36%	2.09%	93.88%	80.80%	13.08%
7	11,887,296	382,122	3	66,238	44,194	134,766	11,259,973	9,842,917	1,417,056
	100.00%	3.21%	0.00%	0.56%	0.37%	1.13%	94.72%	82.80%	11.92%

### miRNA identification

To identify the miRNA sequences in the unannotated reads described above, miRDeep2 software package was used. Our results show that, among the five groups of sequencing results, the miRNA reads accounted for 81.84%, 80.42%, 79.33%, 80.80%, and 82.80% of clean reads (Table 2). In addition, unknown small RNAs make up 12.83%, 13.26%, 13.77%, 13.08% and 11.92% of clean reads (Table 2), and their roles need to be further explored.

**Figure 1:**
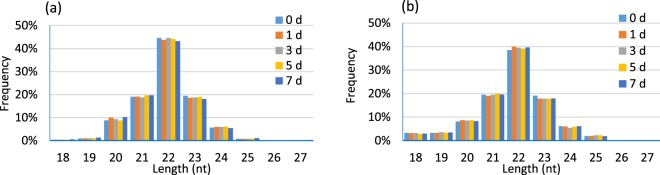
Length distribution of the mapped miRNAs in peripheral blood of dairy cows. (**a**) Known miRNA, (**b**) Predicted miRNA.

**Figure 2:**
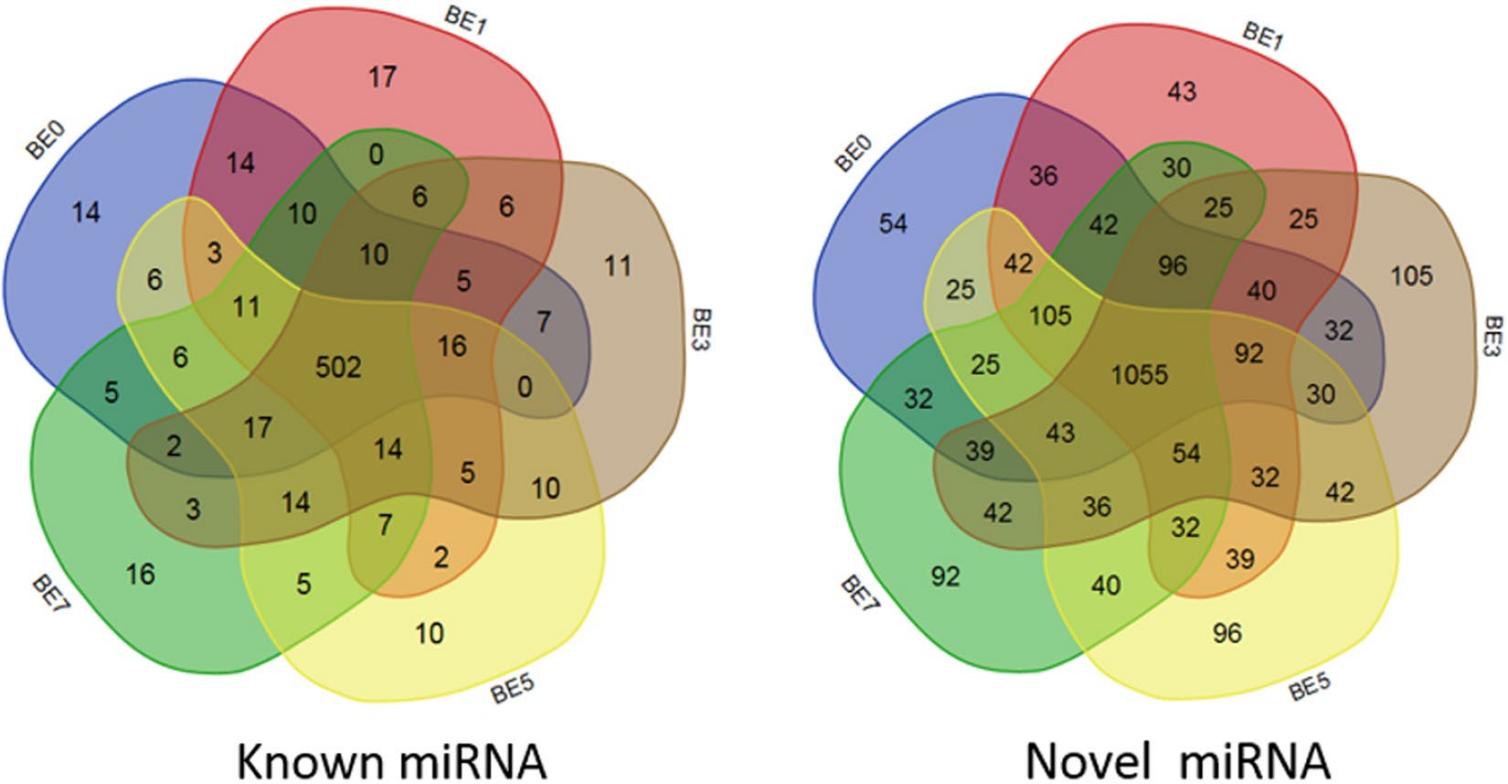
Comparison of miRNA expression in peripheral blood of dairy cows at different times post induction (venn diagram). BE0, BE1, BE3, BE5 and BE7 in the figure represent the groups 0d, 1d, 3d, 5d and 7d after *E*. *coli* induction.

After miRNA sequencing of the five sets of sequencing data, a total of 2416 miRNAs were obtained, including 628 known miRNAs (included in the miRBase database) and 1788 novel miRNAs (Supplementary Table S2). The results of miRNA fragment distribution analysis showed that no matter if the identified miRNA was previously known or newly predicted, the length of the fragment was primarily 22 nt, followed by 21, 23, 20 and 24 nt (Fig. 1). It is noteworthy that, through comparative analysis of the number of differentially expressed miRNA in the five groups, 14, 17, 11, 10, and 16 known miRNAs were specifically expressed in peripheral blood on the 0th, 1st, 3rd, 5th, and 7th day following infection with *E*. *coli*, respectively. Meanwhile, there was specific expression of 54, 43, 105, 96 and 92 novel miRNAs (Fig. 2), respectively, suggesting that miRNAs may play important roles in inducing different activities at different times.

### miRNA expression overall distribution

**Figure 3:**
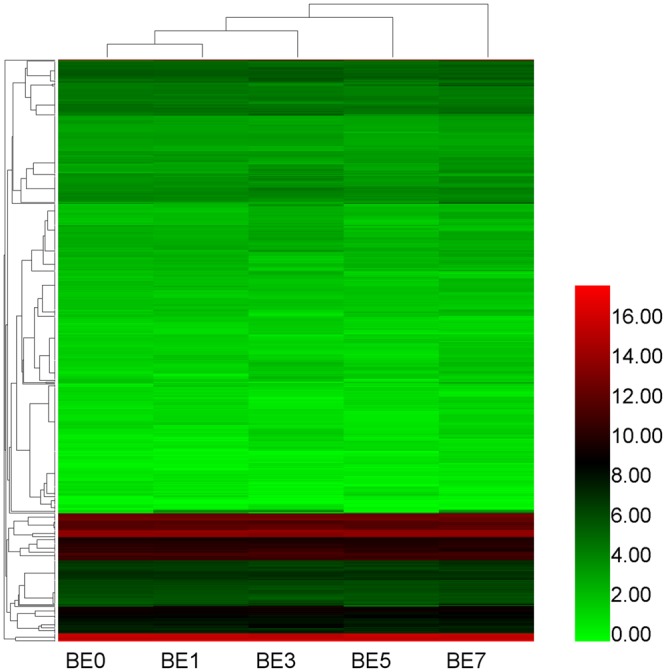
miRNA expression profiles. Heat map is based on the expression of miRNAs TPM-based data, drawn with HemI1.0 software.

Based on our deep sequencing data, we calculated the TPM (transcripts per million) values of the miRNAs from the five sample groups. The overall expression pattern of miRNAs is shown in Fig. 3. As shown in Fig. 3, there are differences in the expression patterns of miRNAs between the five samples, suggesting that these differentially expressed miRNAs may play different roles in the development of mastitis. In addition, the abundance of expressed miRNAs (TPM >1000) (Supplementary Table S3) shows that the top 10 highly expressed miRNAs in all samples are: bta-miR-486, bta-miR-451, bta-miR-92a, bta-let-7f, bta-miR-25, bta-let-7i, bta-let-7g, bta-miR-26a, bta-miR-21-5p and bta-miR-191. In addition, out of all the new miRNAs, unconservative_3_22065 had the highest level of expression in all five samples with a TPM of over 1200.

### miRNA differential expression analysis

**Figure 4:**
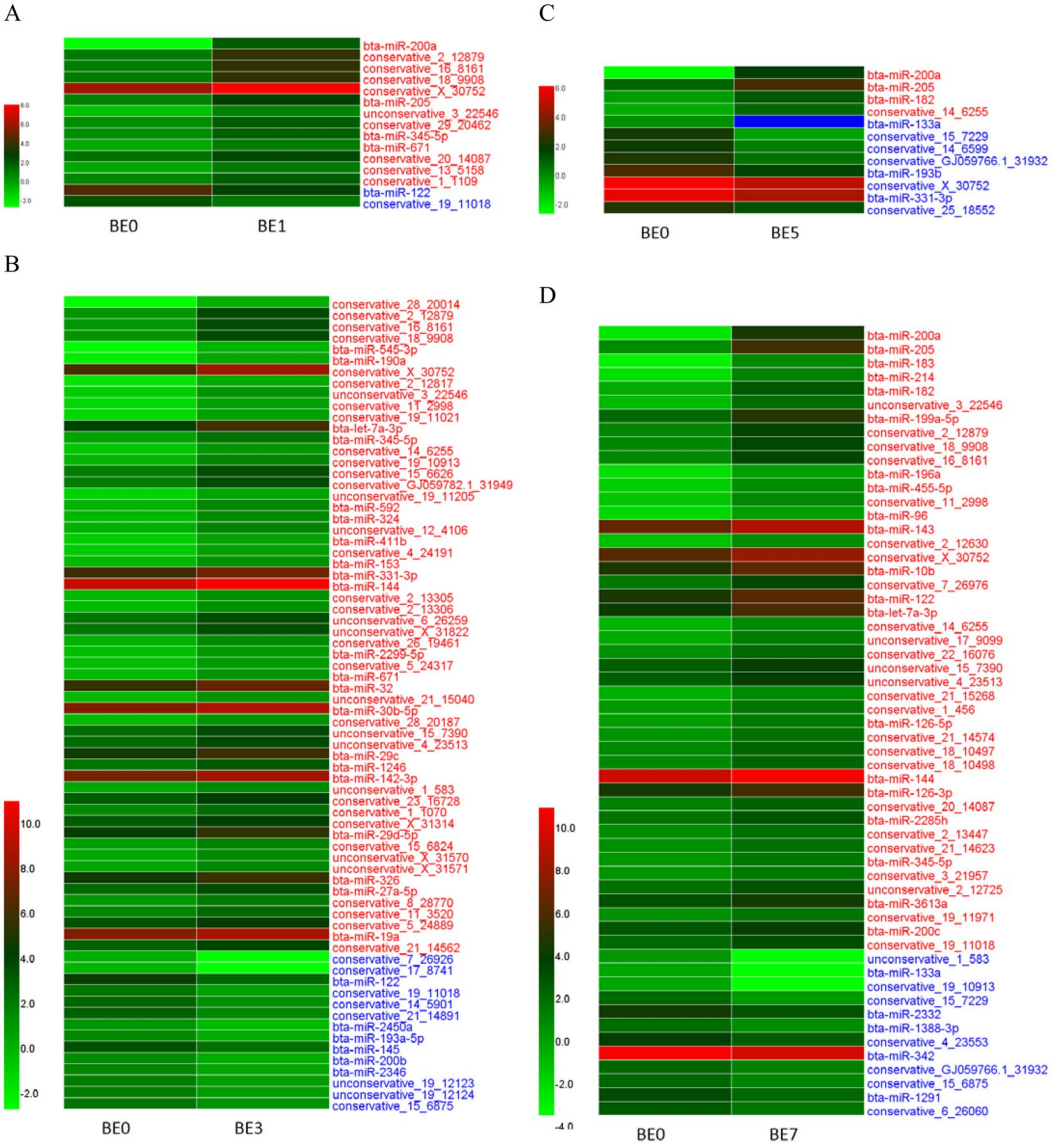
Analysis of differentially expressed miRNA in dairy cow blood with 0 dpi group as control. Based on the sequenced TPM data, heat map was drawn using HemI1.0 software with |log_2_^FC^| ≥ 1 and FDR ≤ 0.01 as the screening standard. BE0, BE1, BE3, BE5 and BE7 represent the 0, 1, 3, 5 and 7 d groups after mammary gland infection with *E*. *coli*; the blue block in the heat map indicates the expression level is 0; the red font indicates that the expression of miRNA is significant increased; blue font indicates a significant down-regulation of expression; from top to bottom, the log_2_^FC^ gradually decreased.

**Figure 5:**
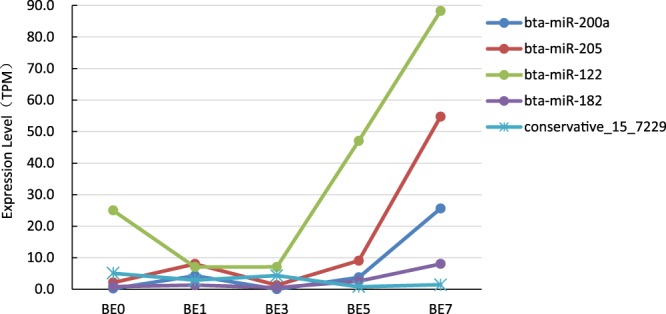
Five miRNAs expression changes.

Based on 5 sets of TPM data for sequencing expression, we performed paired comparative analysis, using |log2(FC)| ≥ 1, FDR ≤ 0.01, TPM > 1 as the selection criteria, and obtained 200 differentially expressed miRNAs (DIE-miRNAs) (Fig. 4 and Supplementary Table S4), of which 76 are known miRNAs and 124 are novel miRNAs (see Supplementary Table S5 for sequence information). Pairwise comparisons revealed that, compared to the control group (BE0), there were fewer miRNAs differentially expressed in BE1 and BE5, while more miRNAs were significantly differentially expressed in BE3 and BE7 (Fig. 4). It is noteworthy that the expression of bta-miR-200a and bta-miR-205 in BE1, BE5 and BE7 groups were significantly increased (bta-miR-200a log_2_^FC^ between 4.36 and 7.13, bta-miR- 205 log_2_^FC^ between 1.93 and 4.70). At the same time, the expression of bta-miR-122 was significantly downregulated in BE1 and BE3 (log_2_^FC^ was -1.82), while it was significantly upregulated in BE5 and BE7 (log_2_^FC^ 0.91 and 1.82, respectively). This indicates that bta-miR-122 plays complex roles in mastitis. The expression level of bta-miR-200a and bta-miR-205 in blood significantly increased on the first day after infection, and decreased to pre-infection level on the 3rd day after infection, while on the 5th and 7th day it was significantly increased (Fig. 5). In addition, our study found that compared with the BE0 group, the expression of the novel miRNA conservative_15_7229 in BE5 and BE7 groups was significantly decreased. The above results indicate that these 4 miRNAs (bta-miR-122, bta-miR-200a, bta-miR-205 and conservative_15_7229) may be involved in the immune process in the late stage of dairy cow mastitis induced by *E*. *coli*, which still needs further study.

Our results showed that, on days 1, 3, 5, and 7 following induction, there were many differentially expressed miRNAs in dairy cow blood following *E*. *coli-*induced mastitis. The differences in expression were very pronounced (Supplementary Table S4). Notably, bta-miR-182 expression significantly increased in BE1_vs_BE7, BE3_vs_BE5, BE3_vs_BE7 and BE5_vs_BE7 comparisons (log_2_^FC^ of 2.65, 2.30, 3.95 and 1.64, respectively) (Supplementary Table S4). This indicates that bta-miR-182 is significantly upregulated at 5 or 7 dpi and may also participate in the immune process in the late stage of mastitis.

### Differentially expressed miRNA qPCR validation

**Figure 6:**
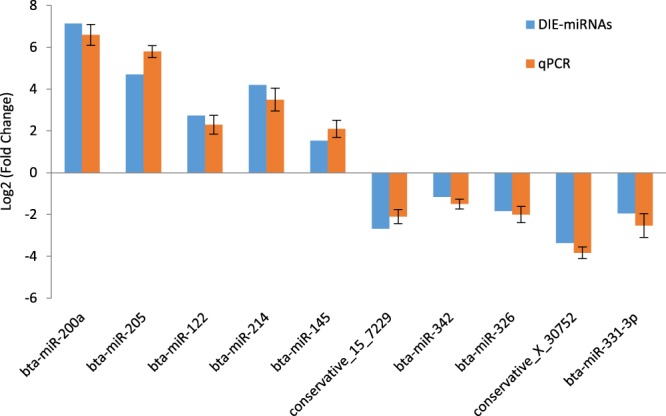
qPCR validation of differentially expressed miRNAs. qPCR expression was normalized with cel-miR-39. There were 10 differentially expressed miRNAs for qPCR validation, with bta-miR-200a, bta-miR-205 and bta-miR-342 from the BE0 vs BE7 comparison; conservative_15_7229 from BE0 vs BE5; bta-miR-326 and conservative_X_30752 from BE3 vs BE5; bta-miR-145 and bta-miR-331-3p from BE3 vs BE7; bta-miR-122 from BE1 vs BE5; bta- miR-214 from BE1 vs BE7 comparison.

Ten differentially expressed miRNAs (five of which were upregulated and five of which were downregulated) were randomly selected to perform qPCR experiments and the qPCR results were compared with the high-throughput sequencing results as shown in Fig. 6. The results show that qPCR results are in complete agreement with the results of high-throughput sequencing, which indicates that our findings of differential expression in miRNAs through high-throughput sequencing are accurate and reliable.

### DIE-miRNA target gene prediction and functional annotation and KEGG signaling pathway analysis

**Figure 7:**
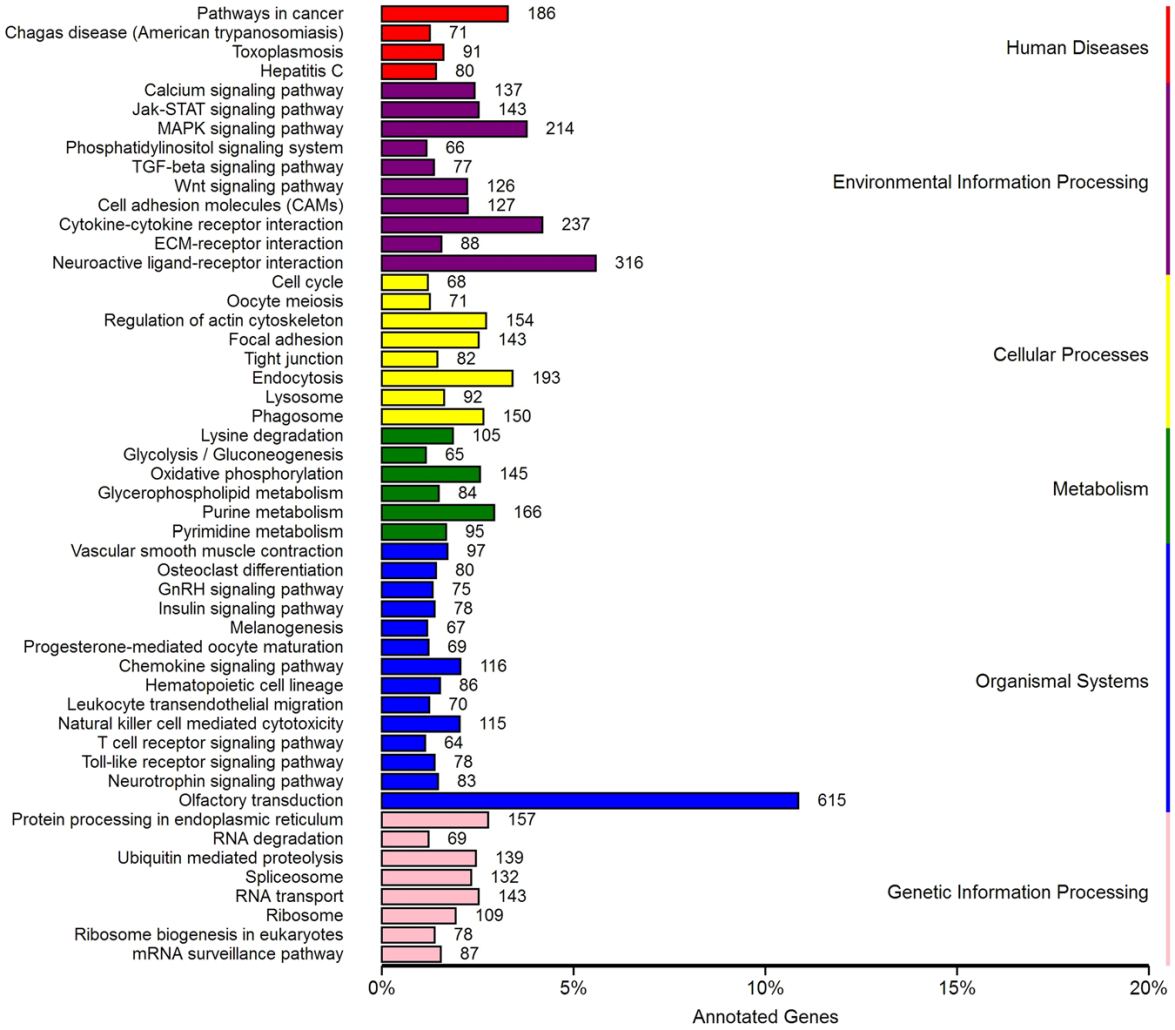
KEGG enrichment pathway analysis of DIE-miRNAs target genes.

To explore the function of DIE-miRNA in the development of mastitis, we predicted the target genes of DIE-miRNAs, and our results show that the 200 DIE-miRNAs may have 18,369 target genes. The results of KEGG pathway enrichment analysis showed that these target genes may be involved in the following 6 aspects: disease, environmental information processing, cellular processes, metabolism, organismal systems, and genetic information processing, and encompass 50 significant signal pathways, of which 7 signal pathways (cytokine-cytokine receptor interaction, chemokine signaling pathway, leukocyte transendothelial migration, T cell receptor signaling pathway, Toll-like receptor signaling pathway, and cell adhesion molecules) are associated with immunity (Fig. 7). Therefore, we conclude that these DIE-miRNAs may participate in the disease development process by regulating their target genes during *E*. *coli*-induced mastitis.

## Discussion

Dairy cow mastitis is an inflammatory disease caused by pathogenic microorganisms. Studies have shown that the expression level of immunity-related genes rapidly changes after pathogens infect tissues or cells, and the process of disease occurrence and development can be finely regulated^18–20^. Therefore, screening differentially expressed genes may play a very important role in the research of disease molecular mechanism or rapid diagnostic technology. In recent years, with the development of sequencing technology, high-throughput sequencing techniques are often used to mine new miRNAs related to immunity and disease and to screen for differentially expressed miRNAs. In the case of dairy cow mastitis, screening of differentially expressed miRNAs based on high-throughput sequencing has focused on both mammary epithelial cells and mammary tissue. For example, Jin *et al*.^21^ found 113 novel miRNAs in *E*. *coli* and *S*. *aureus*-infected bMECs and found 10 DIE-miRNAs. Lawless *et al*.^13^ screened 21 DIE-miRNAs from *S*. *uberis* infected bMECs. In addition, other researchers have also used RNASeq technology to measure the miRNA expression profiles of *E*. *coli* and *S*. *aureus*-infected mammary tissues and the results show that there are multiple differentially expressed miRNAs in infected mammary tissues^10,22,23^.

Although changes in the expression of blood miRNAs have been shown to be putative biomarkers for early diagnosis of disease^7,9^, the differential expression of miRNAs can also be used as biomarkers of animal performance^24^. However, little has been done so far on miRNAs in the blood of dairy cows affected by mastitis. Lawless *et al*.^16^ detected three DIE-miRNAs (bta-miR-146b, -451, and -411a) in blood monocytes in dairy cows infected with *S*. *uberis in vivo*. Li *et al*.^14^ and Chen *et al*.^15^ used qRT-PCR and Illumina’s Genome Analyzer IIe to detect DIE-miRNAs in the peripheral blood of mastitis-affected dairy cows in the clinic. Their results showed that, compared with healthy cows, there were 123 mastitis-related miRNAs that experience significant upregulation (such as bta-miR-16a, bta-miR-125b, bta-miR-15a, bta-miR-29b, bta-miR-301a, bta-miR-21-3p, bta-miR-181a and bta-miR-181b, etc.) and 50 miRNAs that undergo significant downregulation (such as bta-miR-223, bta-let-7f, bta-miR-375, bta-miR-148a, etc.). However, it is surprising that the results of the above two scholars vary greatly, and some findings are even in opposition to one another. For example, their results show that bta-miR-146a and bta-miR-146b are significantly downregulated in mastitis cow blood in one reference^15^, but are significantly upregulated in another reference^14^. Thus it is clear that the specific dairy cow population, feeding environment and the type of infection and time of infection (or the degree of mastitis) have a significant effect on blood miRNA expression profiles. Therefore, we felt that an in-depth study of *E*. *coli*-induced mastitis miRNA expression profile was essential to clarify the molecular mechanism of *E*. *coli*-induced mastitis. Therefore, we used a small dose of *E*. *coli* for a long-term infection of mammary tissue, and used RNASeq technology to detect miRNA expression in dairy cow blood at different times after infection. Our results show that 2416 miRNAs were detected, including 628 known miRNAs and 1788 newly predicted miRNA (Fig. 2 and Supplementary Table S2). The result of comparison of blood miRNA expression at different times revealed that 200 DIE-miRNAs (including 76 known miRNAs and 124 novel miRNAs) were differentially expressed, of which bta-miR-200a, bta-miR-205, bta-miR-122, bta-miR-182 expression were significantly upregulated in late mastitis, while the newly found conservative_15_7229 was significantly downregulated.

As for the function of the differentially expressed conservative_15_7229, thus far, there have been no reports on this miRNA. A closely related gene found in recent years is miR-200a. The expression of miR-200a is significantly increased in many types of disease, consistent with the results of this study. Zhao *et al*.^25^ showed that the expression of miR-200a-3p is increased in hepatocytes of alcoholic liver disease, and may target ZEB2 to induce apoptosis. Wang *et al*.^26^ have shown that the expression of miR-200a in CD4^+^ T cells correlates with the expression of Th17/Treg cells and related cytokines in psoriasis vulgaris. It is noteworthy that, Zarate-Neira *et al*.^27^ found that mouse miR-200a-3p expression was significantly increased in a systemic lupus erythematosus (SLE) model and can inhibit the TLR4/MyD88 signalling pathway and promote interferon production, thereby regulating the immune response in mice and proving its usefulness as a biomarker of SLE. In addition, the expression of miR-200a is significantly increased in fish lymphocytes^28^ and micro-vascular endothelial cells^29^ induced by LPS (cell surface component of *E*. *coli*), targeting the expression TLR1^28^ and ZEB1^30^ genes to regulate the development and progression of disease^30,31^. However, the current relationship between miR-200a and mastitis in dairy cows is unknown. Therefore, in order to explore the function of bovine miR-200a, we predicted its target gene, and the results indicated that ZEB1 gene may be the target gene of bovine miR-200a. In addition, we found that the 3′-UTR of the ZEB1 gene of other species, such as bovine and human, are highly homologous and that the sequences of bovine and human miR-200a are substantially identical (Supplementary Fig. S1). Therefore, we conclude that bovine miR-200a may also target the bovine ZEB1 gene to regulate the development of *E*. *coli*-induced dairy mastitis disease. The specifics remain to be further studied.

As for the expression of miR-122, the results of this study show that the expression level of bta-miR-122 in dairy cow blood is significantly increased in the late stage of *E*. *coli* infection (5 dpi and 7 dpi). Consistent with the trend of our results, miR-122 also significantly increases in the bloodstream late in the murine liver injury model and has been shown to be a biomarker of liver injury^32^. In addition, it has been shown that miR-122 is involved in the regulation of Toll-like receptor signaling pathway after *Vibrio anguillarum* infection of fish (miiuy croaker) by targeting TLR14^33^. Therefore, we hypothesize that bta-miR-122 may also be involved in inflammatory response in the late stage of *E*. *coli*-induced mastitis.

There are only a few studies on the roles of miR-205 and miR-182 in immune regulation. Studies have shown that the expression of miR-205 is significantly upregulated in patients with allergic rhinitis^34^, and that miR-205 can also regulate the expression of erbB2/erbB3 in breast cancer cells to promote apoptosis^35^. IL-2 cytokine stimulation of Th cells can result in a significant increase in the expression of miR-182, reaching its maximum three days after stimulation. Furthermore, *in vitro* and *in vivo* studies using the mouse arthritis model showed that IL-2 induced mouse miR-182 expression shows a dose-dependent increase, while inhibiting miR-182 may be beneficial in the treatment of arthritis^36^. Therefore, we hypothesized that the expression of IL-2 and other inflammatory cytokines in *E*. *coli*-induced dairy cow mastitis was low in early stages, but that during later stages IL-2 and other inflammatory cytokines continued to accumulate and induced a significant increase of miR-182 expression in the late stage of *E*. *coli*-induced mastitis.

In addition, in order to explore the function of DIE-miRNAs, we predicted the target genes of 200 DIE-miRNAs in *E*. *coli*-induced mastitis and analyzed their KEGG signaling pathway. The results indicated that these DIE-miRNAs may be involved in seven animal immunity-related signalling pathways, of which the Toll-like receptor signaling pathway^37^ and chemokine signaling pathway^38^ are closely related to innate immunity and inflammatory response, while the T cell receptor signaling pathway^39^ is related to adaptive immunity. Therefore, we concluded that these DIE-miRNAs may be involved in the innate or adaptive immunity in dairy cow mastitis and may thus regulate the development of mastitis.

In summary, this study used RNASeq technology to detect the miRNA expression profile in the peripheral blood of dairy cows infected with *E*. *coli*-induced mastitis at five different post-induction times. The results show that a total of 2416 miRNAs were detected, of which 628 miRNAs were known, and 1788 miRNAs were unknown. A total of 200 DIE-miRNAs were found to be involved in cytokine-cytokine receptor interaction, MAPK signaling pathway, chemokine signaling pathway, leukocyte transendothelial migration, T cell receptor signaling pathway, Toll-like receptor signaling pathway, and cell adhesion molecules in a total of seven signal transduction pathways that regulate the development of mastitis. In addition, bta-miR-200a, bta-miR-205, bta-miR-122, bta-miR-182 and the newly discovered conservative_15_7229 might be involved in immunity in late stage of dairy cow mastitis caused by *E*. *coli*. The results of this study have laid the foundation for molecular network analysis and molecular breeding of mastitis, and also provide new molecular materials for the biotherapy of mastitis.

## Materials and Methods

### Ethics Statement

All animal procedures were conducted with attention to animal welfare and humanitarian procedures according to guidelines laid down by the Institutional Animal Care and Use Committee of Northwest A&F University, and the protocols were approved by the Experimental Animal Manage Committee of Northwest A&F University.

### Experimental animals and mastitis model induction

Experimental animals and methods used for the induction and verification of mastitis models have been previously described^22^. In brief, healthy 2-year-old half-sibling dairy cows were divided into control and experimental groups. Animals in the experimental group were injected with 5 mL of 10^5^ CFU/mL *E*. *coli* (ATCC 25922) via the nipple. The control group was injected with an equivalent amount of PBS. We adopted a painless surgical method to collect mammary tissue and used hematoxylin-eosin staining and pathology analysis to ensure the reliability of the model.

### Sample collection and RNA extraction

On days 0, 1, 3, 5, and 7 following infection with *E*. *coli*, 5 mL of blood was collected to extract total RNA using TRIzoL according to the instruction manual. The purity, concentration, and integrity of RNA were tested to ensure the use of qualified samples for sequencing (RIN value ≥8.0, 28S/18S ≥1.5; no baseline uplift; 5S peak normal).

### Small RNA library construction and high-throughput sequencing

Based on protocols from previous publications^22,40,41^, 1.5 μg of qualified RNA was taken and a small RNA library was constructed using the method described in the NEBNext® Ultra™ Small RNA Sample Library Prep Kit for Illumina® (NEB, USA). After the library was constructed, the library concentration was tested using Qubit 2.0 and was adjusted to 1 ng/μl. Insert Size was measured with an Agilent 2100 bioanalyzer, and qPCR was used to accurately determine the effective concentration of the library to ensure library quality. After qualification of the library, high-throughput sequencing was performed with the Illumina HiSeq2500 of BioMerker Biotech Co., Ltd. (Beijing).

### High-throughput sequencing quality control

Sequencing reads were single-end (SE) 50 nt. In order to ensure accurate and credible analysis results, the raw data from each sample after sequencing was screened as follows: (1) low quality sequences were removed; (2) sequences with unknown base N (unidentifiable base) content greater than or equal to 10% of reads were removed; (3) reads without 3′ linker sequences and insertions were removed; (4) reads contaminated with 5′ linkers were removed; (5) cleavage of 3′ adapter sequences and T/C/G reads were removed; (7) sequences shorter than 18 nt or longer than 30 nt were removed. This process yielded high-quality, clean, reliable data.

### sRNA classification annotation

Clean reads were aligned using Silva, GtRNAdb, Rfam, and Repbase databases using Bowtie software^17^ to filter out non-coding RNAs such as rRNA, tRNA, snRNA and snoRNA and repeated sequences to obtain the unannotated reads.

UMD3.1 (http://www.ensembl.org/Bos_taurus/Info/Index) was used as the reference genome for sequence alignment and subsequent analysis. Using miRDeep2 software^42^, unannotated reads were aligned with the reference genome for positional information on the reference genome, these are subsequently referred to as mapped reads.

### miRNA identification

To determine miRNA sequences in the unannotated reads, known miRNA identification and novel miRNA prediction were performed using the miRDeep2 software package^42^, i.e. reads and genomic sequences were aligned to obtain possible miRNA precursor sequences using RNAfold and randfold to calculate the energy of the precursor structure and to predict the secondary structure. Combined with information on subsets of sequences (e.g. miRNA production characteristics, mature, star and loop sequences), RNAfold and randfold were scored using the Bayesian model to finally identify miRNAs. MiRNAs with scores greater than 80% were considered to be reliable.

### miRNA expression and differential expression analysis

Expression of miRNA in each sample was statistically analyzed and was normalized using the TPM algorithm^43^. TPM normalization formula is as follows:

$${\rm{TPM}}=({\rm{specific}}\,{\rm{miRNA}}\,{\rm{reads}}\times 1,000,000)/{\rm{total}}\,{\rm{miRNA}}\,{\rm{mapped}}\,{\rm{reads}}$$

The miRNA differential expression analysis between two samples was performed using IDEG6 software^44^. To remove statistical false positives, Benjamini Hochberg’s correction method was used to correct the p-value to the q-value during analysis^45^. Differentially expressed miRNAs were screened using |log_2_^(Fold Change)^| ≥ 1, FDR ≤ 0.01, and q-value < 0.005 as standard.

### miRNA expression qPCR verification

Ten differentially expressed miRNAs (five upregulated and five downregulated miRNAs) were randomly selected from the sequencing data, and qPCR was used to verify the expression results from RNASeq. The specific method used was as follows: using miRcute miRNA isolation kit (Tiangen Biotech Co., Ltd. Beijing, China), miRNA extraction was performed according to the manufacturer’s instructions, and the purity and concentration of the miRNA was measured by a trace UV spectrophotometer and diluted to 50 ng/μl. In addition, at the time of miRNA extraction, exogenous cel-miR-39 (5′-UCACCGGGUGUAAAUCAGCUUG), which has no bovine homologue, was added to each tube for subsequent normalization of qPCR quantification results^46,47^. The reaction system consisted of 4 μl of miRNA template, 10 μl of 2× miRNA RT Reaction Buffer, 2 μl of miRNA RT Enzyme Mix, and 4 μl RNase-free H_2_O. The reaction conditions were 42 °C for 60 min and 95 °C for 3 min to produce the first strand cDNA, which was then diluted to a concentration of 80 ng/μl.

The qPCR reaction was performed using the miRcute Plus miRNA qPCR Detection Kit (SYBR Green) (Tiangen Biotech Co., Ltd. Beijing, China). The reaction system consisted of: 1 μL cDNA template, 10 μL 2× miRcute miRNA Premix (with SYBR & ROX), 0.4 μL Reverse Primer (10 μM), 0.4 μL Forward Primer, and 8.2 μL RNase-Free ddH_2_O. The reaction conditions were as follows: 95 °C for 15 min, 5 cycles of 94 °C for 20 s, 64 °C for 30 s, 72 °C for 34 s, followed by 45 cycles of 94 °C for 20 s, then 60 °C for 34 s. Experiments using qPCR followed the MIQE guidelines^48^: NTC and NRC groups were set up for each qPCR experiment to ensure that the entire experiment was free of DNA or RNA contamination. The qPCR primer sequences are shown in Supplementary Table S1. The qPCR amplification efficiency of each primer ranged from 99.8% to 103.9% (R^2^ > 0.99). To ensure the specificity of primer amplification, primer melting curve detection was performed at the end of qPCR. All quantitative experiments were performed in three technical duplicates.

The miRNA expression level was calculated using the 2^−ΔΔCt^ method^49^, and the exogenous cel-miR-39 was used to normalize the quantitative results. The expression level was expressed as mean ± SD, and Student’s *t*-test was used to test the significance of the difference. A *P* value of less than 0.05 indicated a significant difference.

### miRNA target gene prediction and functional annotation

Using known miRNAs and newly predicted miRNAs and their corresponding species, miRanda^50^ and RNAhybrid^51^ were used to perform target gene analysis. KEGG pathway enrichment analysis was performed using KOBAS software^52^.

## Electronic supplementary material

Supplementary information
