# Supplementary material for: Expression profiling of peripheral blood miRNA using RNAseq technology in dairy cows with Escherichia coli-induced mastitis
Source: Sci Rep. 2018 Aug 23;8:12693. doi: 10.1038/s41598-018-30518-2 (PMC6107498; doi:10.1038/s41598-018-30518-2)
Supplement: Supplementary file 1 — Supplementary information [file 41598_2018_30518_MOESM1_ESM.pdf]

Expression profiling of peripheral blood miRNA using RNAseq technology in dairy cows with *Escherichia coli*-induced mastitis

Zhuo-Ma Luoreng[1, 2], Xing-Ping Wang[1, 2], Chugang Mei[1] and Lin-Sen Zan[1,*]

[1] College of Animal Science and Technology, National Beef Cattle Improvement Center, Northwest A&F University, Yangling Shaanxi, China;

[2] Key Laboratory of Zoology in Hunan Higher Education, College of Life Science, Hunan University of Arts and Science, Changde Hunan, China.

[*] Corresponding author. zanlinsen@163.com; Tel: +86-29-87091923.

List of supplementary information:

Figures S1 Conservative analysis of bta-miR-200a among different species and prediction of ZEB1 target genes.

Table S1 The list of primers used for the qPCR validation.

Table S2 The number of the known and novel miRNA detected by RNAseq.

Table S3 The expression level (TPM) of the most abundant miRNAs.

Table S4 DIE-miRNAs of peripheral blood at different times of mastitis caused by E.coli.

Table S5 The sequences of the novel DIE- miRNAs in peripheral blood.

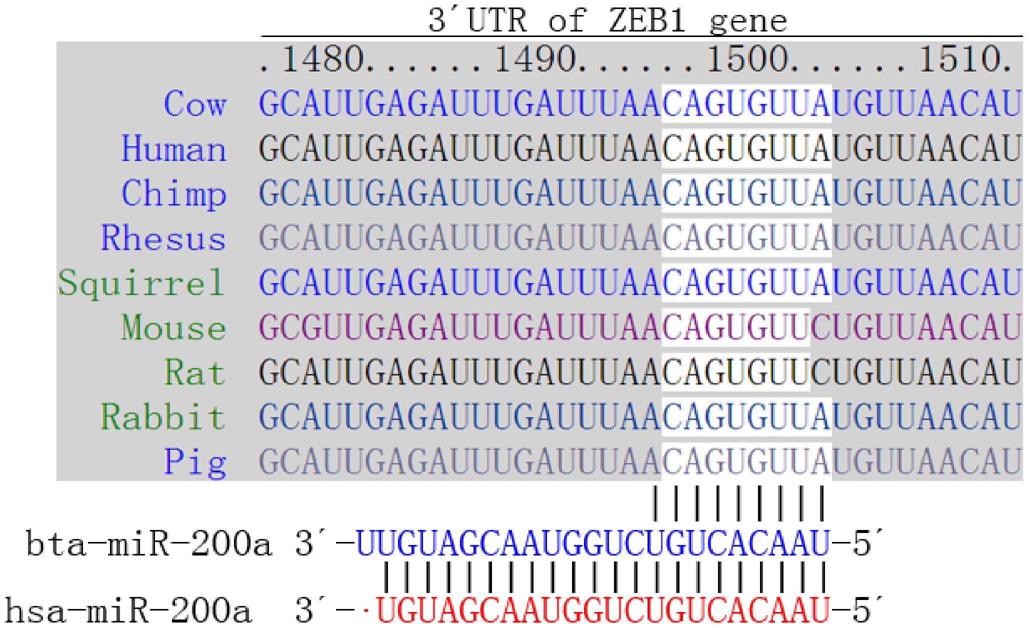

Figure S1 Conservative analysis of bta-miR-200a among different species and prediction of ZEB1 target genes

Table S1 The list of primers used for the qPCR validation

**microRNA name**	**Sequence accession number**	**Primer sequence (5´→3´)**
bta-miR-200a	MIMAT0003822	GGGGTAACACTGTCTGGTAACGATG
bta-miR-205	MIMAT0003545	GTCCTTCATTCCACCGGAGTCTG
bta-miR-122	MIMAT0003849	GGGTGGAGTGTGACAATGGTGTTT
bta-miR-214	MIMAT0003825	ACAGCAGGCACAGACAGGCAGT
bta-miR-145	MIMAT0003542	AGTCCAGTTTTCCCAGGAATCCC
conservative_15_7229	-	GGGGGTGAAAAGTTAGTTTGGGTT
bta-miR-342	MIMAT0003846	TCTCACACAGAAATCGCACCCAT
bta-miR-326	MIMAT0009286	CCTCTCGGCCCTTCCTCCAG
conservative_X_30752	-	CCCTGTGGCCTAGTGGTTAGGATT
bta-miR-331-3p	MIMAT0004339	CCCCCTGGGCCTATCCTAGAA

Table S2 The number of the known and novel miRNA detected by RNAseq

Samples	Known-miRNAs	Novel-miRNAs	Total
0 d	513	1597	2110
1 d	500	1467	1967
3 d	484	1406	1890
5 d	480	1370	1850
7 d	370	1070	1440
Total	628	1788	2416

Table S3 The expression level (TPM) of the most abundant miRNAs

miRNA ID	BE0	BE1	BE3	BE5	BE7
bta-miR-486	150510.3229	150893.0611	112921.0136	126257.277	138342.6275
bta-miR-451	131565.5805	161225.6791	243239.166	127508.3267	138038.3478
bta-miR-92a	74916.67953	63752.91663	43007.97123	71269.37197	65422.37428
bta-let-7f	70904.34711	68249.1957	57174.0416	87197.19368	75932.26683
bta-miR-25	67427.36572	54611.97065	41230.02103	56160.78259	55489.44485
bta-let-7i	60829.48402	57921.59639	50792.55195	68162.86587	55472.37674
bta-let-7g	51460.54483	49146.93842	46890.68742	60764.66934	48872.70715
bta-miR-26a	42617.6676	40747.55714	37804.27764	42854.18925	48434.11765
bta-miR-21-5p	34399.52597	40543.13996	55483.89779	49861.62348	48090.9267
bta-miR-191	24882.73572	21256.09076	15849.40012	23419.45069	20764.2714
bta-let-7a-5p	21994.56307	19770.04004	15545.78664	24883.75092	26466.23963
bta-miR-185	17433.67455	18702.26177	15565.62502	17755.31723	18757.24442
bta-miR-103	16479.67781	17490.06565	15984.12861	14616.32144	13678.16065
bta-miR-101	15345.72808	17717.62864	24179.62257	17892.75844	21156.73636
bta-miR-423-3p	15232.16463	13387.49003	9700.364543	14118.54009	14797.44267
bta-miR-93	13427.76251	13430.11127	11974.10177	11834.61372	8587.90133
bta-miR-148a	12492.43498	14595.49144	13466.37929	14882.34304	17552.01227
bta-miR-30d	10273.57636	10371.56886	9880.031272	11264.96125	10137.13719
bta-miR-16b	8827.076696	8253.015884	9747.459135	7280.839848	5223.55314
bta-miR-20a	8493.854027	9608.206649	9977.153085	9422.894595	6606.171727
bta-miR-16a	7991.925429	7029.883559	7598.876216	6925.717751	5548.456824
bta-miR-199a-3p	7454.25212	6867.113853	6108.927547	6509.062161	7199.390181
bta-miR-151-3p	6834.798555	6502.998094	6166.976375	6578.078127	8659.628035
bta-let-7b	5971.597936	5261.43901	4433.01562	5818.311777	5824.797669
bta-miR-99a-5p	5803.301819	5504.132912	5467.544064	5306.943833	7337.459007
bta-miR-186	5618.704292	5202.638173	6305.241266	5697.115408	4632.163413
bta-miR-142-5p	5316.99161	5954.98927	7087.218493	5708.732932	9547.169807
bta-miR-140	4553.238696	5440.238499	6048.722374	3611.474569	4862.278123
bta-let-7d	4396.326245	3985.198668	3174.485979	4394.279059	5492.985464
bta-miR-30e-5p	4391.681709	4340.40067	8082.070175	5628.690164	5693.73896
bta-miR-423-5p	4055.590357	3841.155343	3191.650491	3781.405464	3818.075475
bta-miR-17-5p	3863.980492	4170.589844	4100.938373	3782.488454	3028.573745
bta-miR-339a	3679.018688	4609.011884	5604.687654	3679.112185	5790.661447
bta-miR-128	3590.726975	3099.590641	2366.373841	4116.836844	5863.200919
bta-miR-30c	3577.339784	4324.146171	4817.017674	3819.507003	4249.350066
bta-miR-181a	3559.53573	2839.668468	2705.782664	2611.776821	2716.06476
bta-miR-339b	3553.160877	4446.391988	5537.668428	3625.159618	5872.141358
bta-miR-148b	3312.555715	3711.269162	3505.183165	3235.578764	3496.422859
bta-miR-221	2960.390625	2697.797403	2539.657786	2622.902077	2520.289463
bta-miR-27b	2814.998444	3065.358816	2642.64486	2549.849514	2894.365563
bta-miR-2284x	2346.947636	2003.348277	2029.983902	1936.975919	2241.510317
bta-miR-150	2145.866561	2344.767662	3471.716679	1759.464097	2240.799145
bta-miR-26b	2097.190006	1871.589458	1922.856644	1834.387279	2036.692984
bta-miR-7	2016.77579	1905.072228	2308.066251	2691.425775	1523.735291
bta-miR-98	1970.330433	1955.408741	1705.842006	2424.813455	2460.144691
bta-miR-15b	1844.336018	1577.285648	1253.440665	1112.427107	1186.233715
bta-miR-320a	1628.77492	1437.736655	1330.120321	1535.974449	1295.144519
bta-miR-425-5p	1479.603362	1379.235439	1543.943567	1152.596172	1199.136394
bta-miR-199b	1446.408592	1652.940229	2366.632602	1599.772375	1734.038802
unconservative_3_22065	1379.336033	1265.22923	1243.003952	1315.832223	1530.643812
bta-miR-22-3p	1308.028749	1142.309262	1336.848119	1129.164218	1731.905288
bta-miR-107	1279.888327	1224.031191	1280.093099	1062.412684	1161.850699

Table S4 DIE-miRNAs of peripheral blood at different times of mastitis caused by *E.coli*

Comparison	ID	PValue	FDR	log2FC	regulated
BE1_vs_BE3	conservative_8_28698	0.0000	0.0001	2.0781	up
	bta-miR-592	0.0000	0.0001	1.9039	up
	conservative_15_6824	0.0000	0.0000	1.7884	up
	unconservative_1_828	0.0003	0.0031	1.7341	up
	conservative_16_7575	0.0000	0.0001	1.5540	up
	conservative_21_15210	0.0000	0.0001	1.5540	up
	conservative_26_19461	0.0002	0.0021	1.4836	up
	bta-miR-144	0.0000	0.0000	1.3833	up
	unconservative_6_26259	0.0000	0.0000	1.3804	up
	unconservative_X_31822	0.0000	0.0000	1.3780	up
	unconservative_1_583	0.0003	0.0031	1.3255	up
	conservative_15_6626	0.0000	0.0000	1.3104	up
	conservative_GJ059782.1_31949	0.0000	0.0000	1.3104	up
	conservative_5_24955	0.0002	0.0021	1.2739	up
	unconservative_21_15048	0.0000	0.0001	1.2717	up
	unconservative_22_15497	0.0000	0.0001	1.2717	up
	unconservative_6_26329	0.0000	0.0001	1.2717	up
	bta-miR-324	0.0010	0.0089	1.1629	up
	bta-miR-147	0.0000	0.0000	1.1604	up
	bta-miR-19a	0.0000	0.0000	1.1217	up
	conservative_23_16675	0.0000	0.0000	1.0806	up
	conservative_8_28986	0.0001	0.0007	1.0742	up
	conservative_6_26445	0.0007	0.0067	1.0723	up
	bta-miR-19b	0.0000	0.0000	1.0637	up
	unconservative_1_1055	0.0001	0.0016	1.0418	up
	unconservative_10_1780	0.0001	0.0016	1.0418	up
	conservative_2_13801	0.0010	0.0089	1.0375	up
	conservative_1_1070	0.0000	0.0004	1.0252	up
	bta-miR-29d-5p	0.0000	0.0000	1.0240	up
	bta-miR-200a	0.0000	0.0000	-25.3476	down
	conservative_18_9441	0.0003	0.0034	-3.7026	down
	conservative_7_26926	0.0005	0.0046	-2.8840	down
	bta-miR-205	0.0000	0.0000	-2.6311	down
	bta-miR-2398	0.0001	0.0014	-1.8410	down
	bta-miR-2450a	0.0005	0.0047	-1.4663	down
	bta-miR-2331-5p	0.0006	0.0059	-1.3815	down
	conservative_9_29482	0.0009	0.0080	-1.3409	down
	unconservative_19_12123	0.0000	0.0002	-1.3371	down
	unconservative_19_12124	0.0000	0.0002	-1.3371	down
	conservative_22_16072	0.0002	0.0018	-1.2490	down
	bta-miR-2346	0.0004	0.0035	-1.2115	down
	conservative_14_5901	0.0000	0.0001	-1.1998	down
	conservative_20_14087	0.0000	0.0001	-1.1783	down
	conservative_21_14891	0.0000	0.0000	-1.1633	down
	bta-miR-2443	0.0002	0.0016	-1.1556	down
	bta-miR-2299-3p	0.0001	0.0006	-1.0558	down
	conservative_26_19293	0.0000	0.0006	-1.0189	down
BE1_vs_BE5	bta-miR-122	0.0000	0.0000	2.7407	up
	conservative_16_7575	0.0002	0.0023	1.3204	up
	conservative_21_15210	0.0002	0.0023	1.3204	up
	conservative_5_24955	0.0002	0.0023	1.3204	up
	conservative_2_12879	0.0000	0.0000	-3.6601	down
	conservative_18_9908	0.0000	0.0000	-3.5847	down
	conservative_16_8161	0.0000	0.0000	-3.5562	down

	conservative_X_30752	0.0000	0.0000	-3.0616	down
	conservative_19_10913	0.0000	0.0003	-2.8785	down
	conservative_15_7229	0.0001	0.0012	-1.8536	down
	bta-miR-331-3p	0.0000	0.0000	-1.5635	down
	bta-miR-345-5p	0.0001	0.0009	-1.4536	down
	conservative_29_20462	0.0001	0.0015	-1.3255	down
	conservative_GJ059766.1_31932	0.0001	0.0015	-1.3255	down
	bta-miR-326	0.0000	0.0000	-1.2634	down
	conservative_14_6599	0.0007	0.0080	-1.2576	down
	conservative_11_2879	0.0002	0.0030	-1.1441	down
	conservative_11_2880	0.0002	0.0030	-1.1441	down
	bta-miR-193b	0.0000	0.0001	-1.1346	down
	bta-miR-500	0.0001	0.0015	-1.0796	down
	conservative_29_21194	0.0002	0.0031	-1.0345	down
	conservative_20_14087	0.0001	0.0010	-1.0319	down
BE1_vs_BE7	bta-miR-214	0.0000	0.0000	4.1947	up
	bta-miR-122	0.0000	0.0000	3.6483	up
	bta-miR-96	0.0006	0.0058	3.0248	up
	bta-miR-205	0.0000	0.0000	2.7724	up
	bta-miR-182	0.0000	0.0000	2.6560	up
	bta-miR-200a	0.0000	0.0000	2.5841	up
	bta-miR-183	0.0000	0.0003	2.5773	up
	bta-miR-141	0.0009	0.0083	2.5552	up
	conservative_19_11018	0.0000	0.0000	2.0387	up
	bta-miR-143	0.0000	0.0000	1.8834	up
	conservative_16_7720	0.0005	0.0046	1.8321	up
	conservative_23_16138	0.0005	0.0046	1.8321	up
	bta-miR-199a-5p	0.0000	0.0000	1.7171	up
	bta-miR-10b	0.0000	0.0000	1.7110	up
	conservative_1_456	0.0001	0.0013	1.4397	up
	conservative_15_7037	0.0007	0.0072	1.4397	up
	conservative_19_11971	0.0002	0.0020	1.3617	up
	bta-miR-126-3p	0.0000	0.0000	1.3587	up
	conservative_3_21957	0.0003	0.0029	1.3573	up
	conservative_21_14623	0.0001	0.0015	1.3328	up
	conservative_16_7575	0.0002	0.0021	1.3272	up
	conservative_21_15210	0.0002	0.0021	1.3272	up
	bta-miR-3613a	0.0000	0.0000	1.2767	up
	bta-miR-126-5p	0.0006	0.0059	1.2698	up
	bta-miR-144	0.0000	0.0000	1.2485	up
	bta-miR-2388-5p	0.0001	0.0007	1.2255	up
	unconservative_3_22546	0.0001	0.0013	1.2084	up
	conservative_5_24955	0.0007	0.0063	1.2053	up
	unconservative_1_1055	0.0000	0.0004	1.2027	up
	unconservative_10_1780	0.0000	0.0004	1.2027	up
	conservative_7_26976	0.0000	0.0000	1.2005	up
	bta-miR-145	0.0000	0.0000	1.1645	up
	unconservative_15_7390	0.0000	0.0000	1.1380	up
	unconservative_4_23513	0.0000	0.0000	1.1380	up
	conservative_19_10913	0.0000	0.0000	-4.4183	down
	unconservative_1_583	0.0004	0.0039	-3.6477	down
	bta-miR-380-3p	0.0008	0.0075	-1.9387	down
	bta-miR-1388-3p	0.0002	0.0026	-1.1950	down
	conservative_29_20462	0.0005	0.0051	-1.1197	down
	conservative_23_16212	0.0001	0.0011	-1.0908	down
	conservative_4_23553	0.0000	0.0000	-1.0604	down
	bta-miR-331-3p	0.0000	0.0000	-1.0079	down

	bta-miR-223	0.0000	0.0000	-1.0026	down
BE3_vs_BE5	bta-miR-200a	0.0000	0.0000	25.1570	up
	conservative_17_9303	0.0004	0.0035	3.7750	up
	bta-miR-205	0.0000	0.0000	2.8075	up
	bta-miR-122	0.0000	0.0000	2.7342	up
	bta-miR-182	0.0000	0.0003	2.3064	up
	conservative_5_25375	0.0009	0.0073	1.7757	up
	conservative_10_2574	0.0006	0.0055	1.5694	up
	conservative_10_2578	0.0006	0.0055	1.5694	up
	conservative_19_11018	0.0000	0.0001	1.5576	up
	unconservative_25_18988	0.0004	0.0038	1.3201	up
	bta-miR-2397-5p	0.0000	0.0001	1.3142	up
	conservative_14_5901	0.0000	0.0001	1.2810	up
	bta-miR-125a	0.0000	0.0000	1.2681	up
	conservative_21_14891	0.0000	0.0002	1.1129	up
	unconservative_19_12123	0.0008	0.0065	1.0890	up
	unconservative_19_12124	0.0008	0.0065	1.0890	up
	conservative_15_6875	0.0003	0.0030	1.0333	up
	conservative_19_10913	0.0000	0.0000	-3.5815	down
	conservative_2_12879	0.0000	0.0000	-3.5681	down
	conservative_18_9908	0.0000	0.0000	-3.5266	down
	conservative_16_8161	0.0000	0.0000	-3.4837	down
	conservative_X_30752	0.0000	0.0000	-3.3583	down
	conservative_2_13305	0.0000	0.0001	-2.6672	down
	conservative_2_13306	0.0000	0.0001	-2.6672	down
	conservative_19_11021	0.0003	0.0030	-2.5459	down
	bta-miR-331-3p	0.0000	0.0000	-2.5008	down
	conservative_15_7229	0.0000	0.0000	-2.4531	down
	unconservative_19_11205	0.0009	0.0073	-2.3939	down
	conservative_11_2998	0.0009	0.0076	-2.1311	down
	bta-miR-324	0.0000	0.0002	-2.1310	down
	conservative_26_19461	0.0000	0.0003	-2.0892	down
	bta-miR-345-5p	0.0000	0.0000	-1.9683	down
	conservative_15_6626	0.0000	0.0000	-1.8795	down
	conservative_GJ059782.1_31949	0.0000	0.0000	-1.8795	down
	conservative_2_13801	0.0000	0.0002	-1.8448	down
	unconservative_12_4106	0.0000	0.0002	-1.8448	down
	bta-miR-326	0.0000	0.0000	-1.8380	down
	bta-miR-30b-5p	0.0000	0.0000	-1.8113	down
	conservative_12_4159	0.0003	0.0023	-1.7634	down
	bta-miR-592	0.0001	0.0010	-1.6351	down
	bta-miR-500	0.0000	0.0000	-1.5621	down
	bta-miR-29d-5p	0.0000	0.0000	-1.5403	down
	conservative_14_6599	0.0001	0.0006	-1.5253	down
	bta-miR-193b	0.0000	0.0000	-1.4872	down
	bta-miR-1246	0.0000	0.0000	-1.3829	down
	bta-miR-29c	0.0000	0.0000	-1.3743	down
	conservative_23_16728	0.0000	0.0000	-1.3699	down
	conservative_GJ059766.1_31932	0.0001	0.0009	-1.3656	down
	conservative_X_31314	0.0000	0.0000	-1.3452	down
	conservative_18_10335	0.0000	0.0000	-1.3412	down
	bta-miR-210	0.0000	0.0000	-1.3371	down
	bta-miR-138	0.0000	0.0001	-1.2731	down
	conservative_24_17568	0.0000	0.0000	-1.2686	down
	conservative_1_201	0.0000	0.0000	-1.2405	down
	bta-miR-2903	0.0000	0.0001	-1.2060	down
	conservative_5_24889	0.0000	0.0000	-1.2034	down

	conservative_19_10772	0.0000	0.0000	-1.1621	down
	conservative_8_28770	0.0003	0.0028	-1.1448	down
	conservative_X_30913	0.0005	0.0040	-1.1311	down
	conservative_11_3520	0.0000	0.0002	-1.1223	down
	bta-miR-23a	0.0000	0.0000	-1.1205	down
	conservative_5_24983	0.0012	0.0096	-1.1140	down
	bta-let-7a-3p	0.0000	0.0000	-1.1046	down
	bta-miR-144	0.0000	0.0000	-1.0948	down
	conservative_1_1070	0.0001	0.0007	-1.0571	down
	conservative_25_18603	0.0000	0.0000	-1.0571	down
	bta-miR-1388-3p	0.0001	0.0005	-1.0436	down
	bta-miR-106b	0.0000	0.0000	-1.0295	down
	bta-miR-652	0.0000	0.0000	-1.0156	down
BE3_vs_BE7	bta-miR-200a	0.0000	0.0000	27.9317	up
	bta-miR-196a	0.0000	0.0005	23.5394	up
	bta-miR-205	0.0000	0.0000	5.4034	up
	bta-miR-214	0.0000	0.0000	4.9903	up
	bta-miR-183	0.0000	0.0000	4.6948	up
	bta-miR-182	0.0000	0.0000	3.9551	up
	unconservative_4_23546	0.0007	0.0056	3.6949	up
	bta-miR-122	0.0000	0.0000	3.6418	up
	conservative_19_11018	0.0000	0.0000	2.6748	up
	conservative_21_14623	0.0000	0.0000	2.5216	up
	bta-miR-10b	0.0000	0.0000	2.4381	up
	bta-miR-143	0.0000	0.0000	2.3977	up
	bta-miR-455-5p	0.0004	0.0036	2.1621	up
	bta-miR-199a-5p	0.0000	0.0000	1.9875	up
	bta-miR-2398	0.0003	0.0029	1.8212	up
	bta-miR-2450a	0.0001	0.0009	1.7309	up
	bta-miR-126-3p	0.0000	0.0000	1.6756	up
	conservative_3_21957	0.0001	0.0009	1.6232	up
	conservative_10_2574	0.0005	0.0042	1.6148	up
	conservative_10_2578	0.0005	0.0042	1.6148	up
	conservative_21_14891	0.0000	0.0000	1.5581	up
	bta-miR-2388-5p	0.0000	0.0001	1.5581	up
	bta-miR-126-5p	0.0002	0.0019	1.5358	up
	bta-miR-145	0.0000	0.0000	1.5308	up
	bta-miR-2285h	0.0000	0.0000	1.5163	up
	unconservative_19_12123	0.0000	0.0001	1.5047	up
	unconservative_19_12124	0.0000	0.0001	1.5047	up
	conservative_29_20691	0.0013	0.0099	1.4206	up
	bta-miR-125a	0.0000	0.0000	1.3799	up
	unconservative_4_23629	0.0000	0.0000	1.3674	up
	bta-miR-100	0.0000	0.0000	1.3308	up
	bta-miR-200b	0.0006	0.0052	1.3293	up
	bta-miR-2397-3p	0.0005	0.0041	1.3236	up
	bta-miR-128	0.0000	0.0000	1.3090	up
	conservative_11_3848	0.0012	0.0093	1.2868	up
	conservative_21_14574	0.0002	0.0020	1.2718	up
	bta-miR-2443	0.0001	0.0009	1.2672	up
	conservative_16_7888	0.0006	0.0048	1.2362	up
	conservative_7_26976	0.0000	0.0000	1.2127	up
	conservative_14_5908	0.0008	0.0067	1.1955	up
	bta-miR-2320-3p	0.0000	0.0001	1.1931	up
	bta-miR-200c	0.0000	0.0000	1.1305	up
	bta-miR-2397-5p	0.0001	0.0014	1.1293	up
	bta-miR-1468	0.0000	0.0000	1.1224	up

	conservative_20_14087	0.0001	0.0007	1.0987	up
	conservative_11_2764	0.0000	0.0000	1.0870	up
	conservative_11_2759	0.0000	0.0000	1.0846	up
	unconservative_19_10937	0.0009	0.0072	1.0186	up
	unconservative_5_24349	0.0009	0.0072	1.0186	up
	unconservative_8_28952	0.0009	0.0072	1.0186	up
	unconservative_9_30254	0.0009	0.0072	1.0186	up
	conservative_26_19299	0.0000	0.0003	1.0111	up
	conservative_26_19301	0.0000	0.0003	1.0111	up
	conservative_14_5901	0.0003	0.0030	1.0099	up
	conservative_19_10913	0.0000	0.0000	-5.1213	down
	unconservative_1_583	0.0000	0.0000	-4.9732	down
	bta-miR-545-3p	0.0012	0.0090	-3.4642	down
	conservative_19_11021	0.0001	0.0012	-3.0857	down
	bta-miR-592	0.0000	0.0000	-3.0492	down
	unconservative_12_4106	0.0000	0.0002	-1.9514	down
	bta-miR-331-3p	0.0000	0.0000	-1.9452	down
	bta-miR-1388-3p	0.0000	0.0000	-1.8378	down
	bta-miR-324	0.0002	0.0016	-1.7231	down
	bta-miR-153	0.0005	0.0044	-1.6706	down
	bta-miR-29d-5p	0.0000	0.0000	-1.6424	down
	conservative_15_7229	0.0000	0.0002	-1.6004	down
	bta-miR-19b	0.0000	0.0000	-1.5992	down
	bta-miR-671	0.0011	0.0085	-1.5711	down
	bta-miR-500	0.0000	0.0000	-1.4643	down
	bta-miR-138	0.0000	0.0000	-1.3853	down
	bta-miR-30b-5p	0.0000	0.0000	-1.3789	down
	conservative_19_10772	0.0000	0.0000	-1.3549	down
	bta-miR-491	0.0001	0.0015	-1.3202	down
	bta-miR-326	0.0000	0.0000	-1.3098	down
	conservative_15_6626	0.0000	0.0000	-1.2735	down
	conservative_GJ059782.1_31949	0.0000	0.0000	-1.2735	down
	bta-miR-19a	0.0000	0.0000	-1.2160	down
	bta-miR-342	0.0000	0.0000	-1.1542	down
	bta-miR-1246	0.0000	0.0000	-1.1491	down
	conservative_8_28770	0.0005	0.0041	-1.0994	down
	conservative_3_22576	0.0000	0.0002	-1.0696	down
	unconservative_21_15048	0.0002	0.0023	-1.0313	down
	unconservative_6_26329	0.0002	0.0023	-1.0313	down
	bta-miR-193b	0.0000	0.0001	-1.0166	down
	conservative_6_26060	0.0001	0.0006	-1.0033	down
BE5_vs_BE7	bta-miR-196a	0.0001	0.0012	23.5394	up
	bta-miR-214	0.0000	0.0000	3.8003	up
	conservative_2_12879	0.0000	0.0000	3.3893	up
	conservative_18_9908	0.0000	0.0000	3.2874	up
	conservative_16_8161	0.0000	0.0000	3.2275	up
	conservative_X_30752	0.0000	0.0000	2.9992	up
	bta-miR-200a	0.0000	0.0000	2.7747	up
	bta-miR-205	0.0000	0.0000	2.5959	up
	conservative_11_2998	0.0001	0.0010	2.5048	up
	bta-miR-199a-5p	0.0000	0.0000	2.2441	up
	bta-miR-455-5p	0.0010	0.0091	1.9712	up
	bta-miR-143	0.0000	0.0000	1.8969	up
	unconservative_3_22546	0.0000	0.0000	1.8684	up
	bta-miR-182	0.0000	0.0000	1.6487	up
	bta-miR-10b	0.0000	0.0000	1.5939	up
	bta-miR-126-5p	0.0002	0.0026	1.5859	up

	conservative_14_6599	0.0003	0.0032	1.4079	up
	bta-miR-126-3p	0.0000	0.0000	1.3862	up
	conservative_1_456	0.0005	0.0051	1.3084	up
	conservative_7_26976	0.0000	0.0000	1.2756	up
	bta-miR-345-5p	0.0007	0.0068	1.2677	up
	bta-miR-100	0.0000	0.0000	1.2153	up
	conservative_21_14623	0.0008	0.0071	1.1608	up
	unconservative_19_11543	0.0011	0.0095	1.1233	up
	conservative_19_10681	0.0001	0.0010	1.1180	up
	conservative_19_11018	0.0000	0.0000	1.1171	up
	bta-miR-2285h	0.0000	0.0001	1.0972	up
	bta-miR-1842	0.0000	0.0000	1.0842	up
	conservative_21_14574	0.0010	0.0087	1.0809	up
	bta-let-7a-3p	0.0000	0.0000	1.0686	up
	bta-miR-22-5p	0.0000	0.0000	1.0660	up
	conservative_18_10497	0.0003	0.0033	1.0454	up
	conservative_18_10498	0.0003	0.0033	1.0454	up
	conservative_19_10752	0.0005	0.0047	1.0454	up
	conservative_2_13447	0.0011	0.0095	1.0453	up
	conservative_22_16076	0.0005	0.0051	1.0173	up
	unconservative_1_583	0.0000	0.0000	-4.4782	down
	conservative_26_19324	0.0000	0.0006	-1.1771	down
	conservative_3_22576	0.0000	0.0001	-1.1423	down
	conservative_15_6875	0.0004	0.0039	-1.0702	down

Table S5 The sequences of the novel DIE- miRNAs in peripheral blood

Name	Mature sequence	Pre-miRNA sequence
>conservative_1_1070	aaaaccugaaugaacuuuuu	auguggcauuccguuaaguauuagacugaccaaaauggucauucagguuuucccaugagaucuuuuggcaaaaccugaaugaacuuuuucuccaauccaauacuuaauaaa
>conservative_1_1109	uggaggugaugaacuggc	acguucacucaccggagggcuggaggugaugaacuggcuaccggggcugauggcuggcgguccucacuuacagcucgggguuugucucuucucccgugaugcggggug
>conservative_1_201	ucccugcccuccaggagccca	cccugauuauugggcuauuaggcaugggguuaugugccagucacgaggugaacacugguaaauaggcugaucccugcccuccaggagcccaggccccagagcaggacugc
>conservative_1_456	uauaaaguaggaagcacu	uuaaaugccucaacaacucuauaaaguaggaagcacuaauauuuccauuuuguaaaugaggaaguugaggcuugaaaagguuaaguaacuugcuugagagaaacccug
>conservative_10_2574	aggugagggcccaggcgucugu	ccucgccugggaagccagguggaaacacaggcgccuggccucuucccuggaggaucugauuagaaguccaggugagggcccaggcgucuguagucuguccuggacccacag
>conservative_11_2759	ucacagugacccggucucc	gacucagcacgaagcccgugggccucgggcuuugcugacggaacggccaccgcagggcugcuguguaaugucacagugacccggucucccucccggcuugcucaaggg
>conservative_11_2764	aacgaagacuucagggcagcu	uauuagaacacaucacgaacaacgaagacuucagggcagcuggaaagucaggaucugcucuugagccuucaguggaaaaugucaagaaugguaucagaacuggcuuuu
>conservative_11_2879	ugccucuccgccaccuccaccu	gccuccuccuccuccucuuaccccgccuguucggcagcggcggaggcggcggugcggcagcgguugucccugccucuccgccaccuccaccucggccuauccccgggcug
>conservative_11_2880	ugccucuccgccaccuccaccu	ggugcggcagcgguugucccugccucuccgccaccuccaccucggccuauccccgggcuggcggcgcuggcggggagggggacaguaguuguagacgcugccccugccuc
>conservative_11_2998	ucacucaguccugucugacucu	cagccuaugggaccgcaaagugucggacacaacugagcgccuaacacuuuucacugcaaaagauguuacgugcuaagucacucaguccugucugacucuuugcgaucccauag
>conservative_11_3520	uaccuguccuccaggagcua	ggcaaccaggguggaaagcaccagucuucuuccugugaugcaagcuuagauggucggauaggacacuauaccuguccuccaggagcuagguguccaggggucaggcaga
>conservative_11_3848	cgcguaccaaaaguaauaaugu	cgcgggcgcgcuccgugccgggcugcugacagcgcauuauuacucacgguacgaguuugaagugucacagcgcguaccaaaaguaauaaugucccgucaccagcagaggugc
>conservative_12_4159	augcaguuucuuccuugacca	cuuuuucucuagugaaaugacccuuaacuugcaggaagguaauuguauuagcaggauuacugaauaacaugcaguuucuuccuugaccauauagguuuccacugaaaca
>conservative_13_5158	ucgcauguggaaaugucugcuucuc	guucaaaaauauguguuggcucgcauguggaaaugucugcuucucauuccuuuggaagcaggaauacguccauaacaugcuacauuaaaaaaggaguucucagggcugccaa
>conservative_14_5901	ugccgcgcuguuucccuggugu	gaggaaagaaggggaggcaguccuggagaggggcgcuggggacacggcugucggcaggucguucugagccugccgcgcuguuucccuggugucugucccuccuccgucu
>conservative_14_5908	uagaaaguucguuuggguuuu	gaaguuaauauuggguuggcuagaaaguucguuuggguuuuuauguaccauguuaugaacuucuuggccaaccugauauuugucucaugggcuuguaaccccaaaguuaua
>conservative_14_6255	cuuccaguuaauagcucuguu	auacggaaaauuggggcuuggagaaauuacagagcuauuaauuggcagaaucuuguuuuaaauccugucucuuccaguuaauagcucuguuaacuucuccaaggauuuga
>conservative_14_6599	ugaaaaguugguuuggguuuuu	cuuguuacugucagguuggcugaaaaguugguuuggguuuuuccguaagggugguauggaaaaccaaacaaacuuuccggccagucgauauuaaggacugaccuuaguuaccu
>conservative_15_6626	ucagccuuagcaucaguucu	guggccaaaguacaggaguuucagccuuagcaucaguucuuccaaugaaaacgcaggacugaucuccuuugggauggacugguuggaucucuuugcaguccaaggucaaaag
>conservative_15_6824	aaaaccugaaugaacuuuu	gaaguaauacuggguuaaccaaaagguucauuuggggguuucccguaagauguuacaaaaaaccugaaugaacuuuucggccaacccaguaccuuuuugugguuuuacuuu
>conservative_15_6875	gcuccuuauuucacuuuccugg	cugcccgccucccucucagauuugccggggacgggaaaucgaaagugaaauaaggaacuggcugcuaccagcuccuuauuucacuuuccuggcugcucgaaaaggugagug
>conservative_15_7037	augcucgucccguccucuagg	gguagauugugagggcuggagcagguuugucuugggagaggguugggagcagagcaggcgugcaccgagcaugcucgucccguccucuaggagucagcgacguaugggg
>conservative_15_7229	ugaaaaguuaguuuggguuuuu	gaggcuuguagugggguuggcugaaaaguuaguuuggguuuuuccauaauggaaaauccugaaugaacuuuuuggccaaccccauguaacuuucucccaauuaacacgacuca
>conservative_16_7575	aaaaauccgaacgaacuuuuug	uuucugggauacaggaauucuauugaguuggucagaaaguucuuuugggucuuucuguaagauguaauggaaaaauccgaacgaacuuuuuggcuaacccaaaacugaaacu
>conservative_16_7720	aaaaacccaaacgaacuuuuug	ugggacuuauuguguuggucauaaaguucgcuuagguuuuuccguaagaccuuauggaaaaacccaaacgaacuuuuuggccaauccaauacaacuccaaccugggugucaa
>conservative_16_7888	uaaaaguucguuuggguuuuu	gcagaguauuggguuggccauaaaaguucguuuggguuuuucuguaagaugguuuggaaaaauccaaaugaacuuucugaccaacccaaugauuuguuaaagcuuagaa
>conservative_16_8161	ucccugugggucuagugguuaagacu	aaaguaagagagggaacuucccuguggucuagugguuaagacuuugccuuccaaugcagguggugaagguuugauaccugguugggcagugaagaucccacgugccucuuggc
>conservative_17_8741	uaaaacccgagcuccuuguuag	uggcggagaagccagugagcucaccagaccccacgaggaguuuggauuuccccugaaagagcaacuggggguaaaacccgagcuccuuguuaggccugggagccaccgcca
>conservative_17_9303	ggcgaaguaguacucccggaccugg	agucggccgcccggaccucggcgaaguaguacucccggaccugggucuugaagucguccaggaccgggagcugcuucugcacauagaggucgcugacggagcgcagcguguacc
>conservative_18_10335	ucccuguccuccgggagcua	agacagaaggagcagacaucccuguccuccgggagcuaacacucuaaggugggggagggacaguagacaagaaggaugaaucgaguaucuagucuguuuaaugcugagau
>conservative_18_10497	gcccugcccuccaggagcucc	ggagucaguccugccaccugcccugcccuccaggagcuccuacccaguguggggaccagucuaggcucgggcauggccccagggcccuugcugucucucuucuuuguccu
>conservative_18_10498	gcccugcccuccaggagcucc	aaaccgaugacaugccagcuccaugguggggugacgccgggcacccagaggggagucaguccugccaccugcccugcccuccaggagcuccuacccaguguggggaccagu
>conservative_18_9441	caagacggacagaggugugugca	ugaaccugggcagcaggcccuccucacaccucugacgucucucccuguagacgagcuggagccacugcugcaagacggacagaggugugugcaggcucgcugcagccuca
>conservative_18_9908	ucccugugggucuagugguuaagacu	auuaggcgugcauagggacuucccuguggucuagugguuaagacuccacacuuccacugcagggggugcggguuugagcccuguccagggaacuaagauccugcaugcuuuga
>conservative_19_10681	gaaccagauugucagcucu	cuguucaugugucaguguuguuagagcugacaguuugguuccuuguauccuguuagccacuuuguuuuaggaaccagauugucagcucuaacaacacugaugacugau

>conservative_19_10752	ggcugguccgaagguggua	ccucacggugccuagagcagggcugguccgaaggugguaucggaaagacacagaauuuggaguccguggacccagaucuaucgcuuaacggugcugugacuuaaugau
>conservative_19_10772	gagcacuguucgugaccugcuggu	gaguaaaagaaagucuuugggagcacuguucgugaccugcuggucugccuguggcuggcgguuccauuccggugccauagcagcuccagcaccacgugacugacugccugg
>conservative_19_10913	uuccccugcaucugcaugccagg	auggauuccugacuuuggaucugggugcugguagcagugggaggagagcagaguuaaugggucuugagccuuccccugcaucugcaugccaggugcugggcauugagg
>conservative_19_11018	ugcgaccucagaccagac	uugcuagcugucaugaaggcugcgaccucagaccagacacacuuuagggcugcuuggcugggguaggggaaucaugcacagcauguaauagcagguggcagguucuga
>conservative_19_11021	agaaaguucauucagguuuuu	guacaguuaugcauauauauauugaguuggccagaaaguucauucagguuuuuccacaccaucuuauaggaaaacccgaacuaacuuuugagccaauccaauacauucuuuu
>conservative_19_11971	acuggacuuggagucaga	cauuguaguggaaagagcacuggacuuggagucagacuuccugguucaaguccuggcucugccacuagcuagcuguggguaagugaccuugaguaaauuacaggaca
>conservative_2_12630	ugccugccugagcgucgcc	cccccaucacuggcagcugugccugccugagcgucgccggcucugccaggaggaagcccagccagggcagagcugucaggucacaccugugugaagugagauggagca
>conservative_2_12817	gcguugguguauaguggugagc	gguauguauuccuguguuuuacaggaccauccauuaucuuucucaucacuuaaacucagcaugugcuggugcguugguguauaguggugagcguagaagccuuccaacaugugc
>conservative_2_12879	ucccuguggucuaguaguuaggacu	ccacgcuggccucacucaugccaguguucuguccuaccucugggccuuaaaacauaccauaccugggaauucccuguggucuaguaguuaggacucugagcucucacugccaa
>conservative_2_13305	gcauggugguucagugguagaag	aagcaucacuucauuggugcauggugguucagugguagaagucuugccugccacucaggagguccgauuucuuuuccuggcccaugcagccgcagugucccuuuuggggg
>conservative_2_13306	gcauggugguucagugguagaag	aggcccucagggauccaaccccccuugagagaugccacugacauuuggaaagcaucacuucauuggugcauggugguucagugguagaagucuugccugccacucaggag
>conservative_2_13447	gaaaguuuguucggauuuuuca	uauuuguauugaguuggccagaaaguuuguucggauuuuucauaccaccuuuuggaaaaaacugcaacuaacuuuuucaccaacucaauauuaugacaaugugauuaugu
>conservative_2_13801	ucccuguccucccgggagcuga	ggacacccaggcccccaucccuguccucccgggagcugacccaccagcaggaaugaggacauaggaucggggcuuagggagcugguccugcuaccuuugugaccauggg
>conservative_20_14087	ccugucugagcgucccug	ggggcuccgggcgguccaggccugucugagcgucccugggccugcgccugcacacgcgcccagaggccgucugcccaggaauuaauggcccacacccuuuguaggugu
>conservative_21_14562	aaaaucugaacaaacuuuuugg	uucuccauguugauuggccaaaauguuugcuuggguuuuucaagauguggaaaaaucugaacaaacuuuuuggcccacccucucugcaugcucuagcgauggguccagucc
>conservative_21_14574	cgccugccugagccucgcu	uggccacgacccccgcugcccgccugccugagccucgcugggaagugcuuccaggcugucguaguggacggaccgacugccccgcgaacgcuggucacgggaucauggg
>conservative_21_14623	uuccacucggcaggcuguca	ccgccuccugccucccaggacgccccgcccaccgcauccugccugggucggagccgcggggagcucgagguuccacucggcaggcugucacacguaagucacaauucg
>conservative_21_14891	cccguuucucucgcgcgccggcu	gccuuuagggcggagccggcucccgcucucccucagucggccgcggguagcggacgaucggcccgcgucgcccguuucucucgcgcgccggcucgcuagcucgcucucucu
>conservative_21_15210	aaaaaauccgaacgaacuuuuug	uaccugagacaagacagaacauaguggguuggccaaaaaguuuguucggguuuuccacacagcuuccggaaaaauccgaacgaacuuuuuggcuaagccagcacuugcuua
>conservative_21_15268	gagcucaucagacugauguua	gcgccucuggcuccaggaugugugcccaccccuugcacagauggaaacgcugagucuaggggagaaaggagcucaucagacugauguuagugguggcaaaagcuugugug
>conservative_22_16072	uuccuaccucggacauuc	aaaaggaaagugcaaacaggaacagggaaucuugagaaagucugaggggaggaggcucagaugcaagcucuuccuaccucggacauucagccaggaggaaccacuccc
>conservative_22_16076	gcccuguccucccgggagcucc	guuugcugagcaccugcccugugcuggggccacacuaggcgauggggacacgggggugaacaggacagggcccuguccucccgggagcuccagggcugggcagaaggauag
>conservative_23_16138	aaaaacccaaacgaacuuuuug	cauauauuauuggguuggcuaaaaaguucucuuggguuugcccauaagacauuacggaaaaacccaaacgaacuuuuuggccagcccaauaugaugaauguucacauuggu
>conservative_23_16212	aaaaaggucguuuggguuuucg	auguuaauccugggguuggccaaaaaggucguuuggguuuucguguaacagcuuauagaagaacccgaacaaacuuucuggccaacccaauaacuuuucugcauaacacaua
>conservative_23_16675	uugcaugacucugagaguaagu	augggacaucuggggcacacaguuuaaggaggcacuugcuuucagggaccugcaaguacugguccaucauuugcaugacucugagaguaagugccuccuuaaaucgug
>conservative_23_16728	ucccuguccuccaagagcuug	uuagaagcaagggaguggggucccuguccuccaagagcuugcagucucuuggcuuccaggacgggguaggcugcugggaaggagagaagccacccccaauuugaaccac
>conservative_24_17568	ucccugcccuccaggagcccc	aauacgacccccacccaugguccccugcccuccaggagccccugggugccuggacaaaagaugcaguugaguucuggggcauggagaaaggggcaccagacccaggcuugg
>conservative_25_18552	uccaaguaauucaggauagu	ucauaaugacauggaacccguccaaguaauucaggauaguccuugggacuucuugguggugcaguggcuaaggccccacacucccaaugcaggggcccagguuccauc
>conservative_25_18603	ucccuguccuccaggaggccu	aagcccagagucccuccacucccuguccuccaggaggccuguggccaugugccugcugucuggggucugcuugcccucugguccaucugagguuggggccaugaccugg
>conservative_26_19293	ucgagccugacagaucacaca	cccggacugcugugagcagggcaguggccccucugugaccugccaggcacugccuugaaaucccccuaaggucgagccugacagaucacacacgggacuugaccuggagc
>conservative_26_19328	aaaaguugguucgaguuuuucu	gaaaaauacuggguuagccaaaaaguugguucgaguuuuucuguaacaucuuaucagaaaaauccaagcgaacuuuuuggcuaacucaguacaaaggaaauaagucccuca
>conservative_26_19324	accuuuguucggguuuuucugu	gauauuuuggguugaucaaaaccuuuguucggguuuuucuguaacaucugacggaaaaacccaagcgaaguuuuuggccaccccaaucauuccuaagguauuuaaaaac
>conservative_26_19461	ucccuguccuccaggacccu	aaagcccagcugcauggcucccuguccuccaggacccuucagacggcaccuggagugacgucagagcagggucugggggguguccucacccagaggaucgcccugcacc
>conservative_28_20014	agaggaacaauucugagg	agacugaggaauaaagcaucagaggaacaauucugaggcuuugacagaauucagagcaguuaaauaguucuucgauguuuuacccagcuuuuaaccacaggcucuuuu
>conservative_28_20187	aaaaucugaacaaacuuucugga	uuuaccaacugauugggaaguauuggguuggccaaaaaguucguuaugauuuuuccaugagauguuacagaaaaaucugaacaaacuuucuggacaacccaauaugucau
>conservative_29_20462	uagcacuuguguuuguuuuuguuu	auauauaaugcauuuuacaguagcacuuguguuuguuuuuguuuaauuuguggacaaagacuuacagacaagugcgaaaguaaauccucuuuugcaacccacaacucauugu
>conservative_29_20691	uuucucucgugcucuguguagg	guucucugccgucugggaagauugcucaggcucugcggagcucgagggaaaccuuacucugggggguuccuuuucucucgugcucuguguaggauaucgccauugggcuc
>conservative_29_21194	caaggcacugucuccccugcag	agaggccaagacaucugcccuggacuuccuggagggcccgggagagguggugcccggguccgggggugccaaggcacugucuccccugcagucucuuccccacgaugccga

>conservative_3_21957	ccggccccgcgagcuccccgcga	cugggcggagacaggcggcggccgcgcggggagccgaggcggccagcgcagcggguucagagcaccggccccggccccgcgagcuccccgcgagcugccgccacagaccc
>conservative_3_22576	auugauuaucgaggcgauucugauc	uggggcccggcgcucagaagugaugaauugaucagauagacgaggccgggcuugucccgcgccauugauuaucgaggcgauucugaucugagcccggcccagggcacg
>conservative_4_23553	uccuguccugagcgucgcg	aaggcacgcgccguaguggguggagggggcaaaggaagaaaggaggagggccgagggacggaaacgcgcuuccuguccugagcgucgcggaagcugccaagggguuuc
>conservative_4_24191	accgggugcugcaggcuug	cgcgucacuuccuccccagcaccgggugcugcaggcuugccgguccuccucgcagcgucccaaugcguguggugauaacauaagugaaagucgcucagucgugucuga
>conservative_5_24317	aaaaguucauucagguuuuucc	uaaugaacuauggauaugaauguauuggaugggccaaaaaguucauucagguuuuuccuguuacaagaaaacuugacgaacuuuuugaccaacccaauacauggaagcagg
>conservative_5_24889	ucccugcccuccaggagcuuc	ggaguuuaggggauccagcugugugcuguacaccugccaggcccuggggauacagagacaugucauucagucccugcccuccaggagcuuccgaaaggcagguaccgcu
>conservative_5_24955	aaaaaauccgaacgaacuuuuug	gcuggaggacauccuuaaguaguuuuguuuuuuagagaguucauuugggguuuucccaucacauguuaaggaaaaaauccgaacgaacuuuuugaccaacucaauaaaaagaaa
>conservative_5_24983	uccauccccucuugcccaccagu	cagccccgaagcuggggucccgcaggggccgugggacagaguggaaggaggauccgugcucaccaggcccuccauccccucuugcccaccaguccuggacaugcagcaga
>conservative_5_25375	aaguucguuuguguuuuucugu	uauuuauugggguuggcccaaaguucguuuguguuuuucuguaagacaaauaaaaaaacucgaaugacuuuuuggccaacccaauaaaauaaaucaauugcauggauuuuua
>conservative_6_26060	cacccuuuccagugcccuuuu	acccugugagaaacccagaucacccuuuccagugcccuuuuaagguugacacagugccuuaagaggcuaacacaggaggguaaagaaagucuccauaaaacccagagaa
>conservative_6_26445	aaaaccugaaugaacauuuugg	uuuaauauuggguuggccaaaaaguucguuuagguuuuuccauaccaucguauaggaaaaccugaaugaacauuuuggccaacccaauauuaagcuagaaaaguaauggc
>conservative_7_26926	ugggcguuuggcacuguucaug	ugucuucgucagcagagcccugggcguuuggcacuguucaugacucuggcuuuaugauaugggcgggaccaaaggcccagugaaaugcugucccuugucccagugguaa
>conservative_7_26976	aucugugggauuaugacu	ugaggagccaaugaggccaagcuacuaucugugggauuaugacugaaugcccucuaagucagaaucccgcccagacugaaggauucggccgugccacaggagccucaga
>conservative_8_28698	aaaaccugaaugaacuuuu	uguauuuauuggguugcccgaaaaguucguuuugguuuuuccauaugcucauacagaaaaccugaaugaacuuuucuggccaacccaauauuuauuuaucccugccccccu
>conservative_8_28770	aaaaccugaaugaacuuucugu	guaaaauauuggguuggccagaaaguugguuuggauuuuuccauugcaucuuacagaaaaaccugaaugaacuuucuguccaagccaauauaaaugcaagauaacaaggag
>conservative_8_28986	aaaaacuugaacgaacuuuuug	uguuaauuaauacacaaguaauuacuggguuggccaaaaaguucauuggggguuuucuguaaauuguaauggaaaaacuugaacgaacuuuuugcccaaugcaauacaugu
>conservative_9_29482	cuggcccucucugcccugccu	auagaaaggauccuacuccccuggcccucucugcccugccuucgacucuagcucacggacacaccuuugcuugggcagguagagagguuuguccauuaaccuucuauggu
>conservative_GJ059766.1_3193[illegible]	cacgcucaugcacacgcc	gacacacacaggcgugugcacacacgcacaugugcaugguauacacgugcugcacguauggacaugugcacgcucaugcacacgccugcguacucgcucuugcacacg
>conservative_GJ059782.1_3194[illegible]	ucagccuuagcaucaguucu	guggccaaaggauugcaguuucagccuuagcaucaguucuucccaaugaauauucagggcugauuuccuuuaggagugacuugcuggaucuccaacacaaauucagguuuuau
>conservative_X_30752	ucccuguggccuagugguuaggauu	gaaaugaugccuuugggaguucccuguggccuagugguuaggauucugugcuuucauugucagguucauucccuggucagggaacugagauucuguaaaccauguggcauagc
>conservative_X_30913	ccccucugucuacuccaccaga	aaaagggacugggagguugagguuggugggggugaccaagggagagcuggucugagacuaacuccuucuuccccucugucuacuccaccagaggccagagaaugcguga
>conservative_X_31314	ucccuguccuccaggaucuug	acucuugaaggcauguucugugacugacacccagcuaaguguagggcuacggagugaguaagcauugguccccuguccuccaggaucuuggugucuagugcaggauagcu
>unconservative_1_1055	aaaaacucaaaugaacuuuuug	uuauaaguauuggguugaccaaaaaaguuuguuuggauuuuuugugacaucuuacagaaaaacucaaaugaacuuuuuggucaacccaauauauugauuaccaauaaug
>unconservative_1_583	uaaaaagcguaagauuucccu	caccuucacuagcuugcuggggaggggaaaucuuacacuuuuuauguauaaagcgcagauuugugguuuauaaaaagcguaagauuucccuucuccaacaaagaauuuca
>unconservative_1_828	acaaccugaaugaacuuuuugg	aacuuuuuguugugaagauaaaaagcauuuaauauuggguuggccaaaaguucaguuggguuuggccauacaaccugaaugaacuuuuuggcuagcccacuaugauacccu
>unconservative_10_1780	aaaaacucaaaugaacuuuuug	ggaugacacgggguuggccaaaaaguuaguucggguuuuccuguaacgucuuauggaaaaacucaaaugaacuuuuuggucaauccaauauauaggaaaguuauuaaga
>unconservative_12_4106	uccacaucccucacaguuuggu	aggucaggcuggccuccggguccacaucccucacaguuuggugcuggaaugaaaaccccaggcuguuggaauguugacccagggaccagggcugggggauugggaaucagc
>unconservative_15_7390	aaaaacuagaaugaacuuuuu	cauucuccugcucuauuaauuauugaguuggccaaaaagucuguucagguuuuuccaaaucauugaagggaaaaacuagaaugaacuuuuuggccaacccaauacuaugccu
>unconservative_17_9099	uuaggcucuagagugugugg	uccacgcugcuguggagcuuaggcucuagagugugugggcuucaguagucgguggcucccaggcucuagagcccaggcucaauaguugguggugcacugucuucaugcuu
>unconservative_19_10937	uggcacgccucaggcuucuga	aaguugauucccucuuccaaggggccuacuagugccccucuucaggaaaguagcaacuuggaauaaaaucuggcacgccucaggcuucugaggaaaagggggguguuuauu
>unconservative_19_11205	uccuccccugguccuccacaga	uuguaagcggcuuccuaagacugcuuggcuggaguggugggagggguaggaggaguugcaguucauguuuuuccuccccugguccuccacagacaucaaggagcaugugaa
>unconservative_19_11543	aggaacccagaugaacuuucu	uguaucuugagggucucagauauuggguuggcuggacaguucacuuggguuuuuccauaacaucuuacggaggaacccagaugaacuuucuccccaacccaguacaagc
>unconservative_19_12123	agggagucccugguaguucagu	uuguaguuuaaaaaacaacagggagucccugguaguucagugguuaagacuccaugcuuuucauugcaggaggcauggguucagucccuggucagggaacuaagaacuggc
>unconservative_19_12124	agggagucccugguaguucagu	ugugggugagugcagggaguccuaaccacuggaccaccagggaauucccauuguaguuuaaaaaacaacagggagucccugguaguucagugguuaagacuccaugcuuuu
>unconservative_2_12725	auagcuguacuuucuccuuu	aaccccgguucaauuccuggguugggaagauccgcuggagaaaggauaggcuacccacuccaguagaaggauagcuguacuuucuccuuuuuaguauguguuccuugc
>unconservative_21_15040	aaacccggaugaacuuuuuggu	cacagcagggggaaaucucuuggguuggccaaagaguucauccgggucuccccauaagaugcugcagaaaaacccggaugaacuuuuuggucagcccaauacuugaaguag
>unconservative_21_15048	aaaaaccuuaaugaacuuuuugg	agguguggcgucaguguuauuggguuggcugaaaauuucauucacguuuuuuccauaagaugcuauacaaaaaccuuaaugaacuuuuuggccaacccaguguauggccug
>unconservative_22_15497	aaaaaccuuaaugaacuuuuugg	uggugcacagaacugaaaguauuagguuagccagaaagcucuuucaggauuuuccauaaaauguuauggaaaaaccuuaaugaacuuuuuggccaauccaguauuuccuagc

>unconservative_25_18988	ucugaacgaacuuuguggca	uugaaaguaagcuuuacccugaguauuggguuggccaaaaguuuguucaaauuuuccuguaacaguaaaaucugaacgaacuuuguggcaaacccaauacuauggcaugcu
>unconservative_3_22546	gcuaccaucugugggauc	uuggaguaggaaaugcaacccacuccaguguucuugccuggaaaguuccauggacaaaggagccuggggggcuaccaucugugggaucccaaagagccagacaugacu
>unconservative_4_23513	aaaaacuagaaugaacuuuuu	aaugagaucuuguuauacuguauuaggucagccaaaaaguucguucaaguuuuuccauaacaucuuacggaaaaacuagaaugaacuuuuuggccaacccaauauauuaaa
>unconservative_4_23546	acuacugaagccccugugcccu	cuaucaagccugugcuccagagccugggagcuucaauuacugagcccacgugccucaacuacugaagccccugugcccuagagccugugcucugcaacaagagaaggcacc
>unconservative_4_23629	acacgcguccuuggauccugacu	cguguaaguggacgugcaguccagaggcgagcucggggcccagggccgguguguuuguugccuuaggaacacacgcguccuuggauccugacucgccugggaaacgucauc
>unconservative_5_24349	uggcacgccucaggcuucuga	aaguugauucccucuuccaaggggccuacuagugccccucuucaggaaaguagcaacuuggaauaaaaucuggcacgccucaggcuucugaggaaaagggggguguuuauu
>unconservative_6_26259	aaaacacgaacaaacuuuuugg	cugguaagcccgaguguuacauggggguuggccaaaacguucguucagguuuuucuagaauauuuugcaggaaaacacgaacaaacuuuuuggccagcccaauauuacuu
>unconservative_6_26329	aaaaaccuuaaugaacuuuuugg	uuuucucuagcaaauguuguauugaguuggccaaaaaauucauucagauuuuuccauaacauguuauggaaaaaccuuaaugaacuuuuuggccagcccaauguuagaaacu
>unconservative_8_28952	uggcacgccucaggcuucuga	aguugauucccucuuccaaggggccuacuagugccccucuucaggaaaguagcaacuuggaauaaaaucuggcacgccucaggcuucugaggaaaagggggguguuuauuucc
>unconservative_9_30254	uggcacgccucaggcuucuga	aguugauucccucuuccaaggggccuacuagugccccucuucaggaaaguagcaacuuggaauaaaaucuggcacgccucaggcuucugaggaaaagggggguguuuauuucc
>unconservative_X_31570	uuacaauacaaccugauaagugc	ugauaacaaaggaagggagacaggcauauacacagacaauuacaauacaaccugauaagugcuguaacacuuaucagguuguauuauaauggccgauguaaaauucauu
>unconservative_X_31571	ugauaauacaaccugauaagugc	ccauagacacggacaaugauaauacaaccugauaagugcuaggacacuuagcagguuguauuauauccauccgaguaggauuucuucagguugcuguuggauccaugaa
>unconservative_X_31822	aaaacacgaacaaacuuuuugg	acuaagccauuaaaaaucuauuggguuagucaaaaguucguuggaguuucucuguuacauuguacagaaaaacacgaacaaacuuuuuggcugacccacuacuucugca
